# The state value

**DOI:** 10.1371/journal.pone.0320029

**Published:** 2025-06-17

**Authors:** Moustafa Ahmed AbdElaal, Nesrien Mohamed Elmohamady

**Affiliations:** 1 Department of business administration, Faculty of Business, Alexandria University, Egypt,; 2 Department of public finance, Faculty of Business, Alexandria University, Egypt; Instituto Superior de Contabilidade e Administracao de Lisboa—Instituto Politecnico de Lisboa (ISCAL-IPL), PORTUGAL

## Abstract

This paper considers the state value using the future contract pricing approach. Our model allows adding an unlimited number of factors that affect the state value. We think this model may be useful to evaluate the performance of the government and the decision-making process by knowing the optimal value and the optimal time for the decision. This dynamic model differs from the traditional pricing model for evaluating the nation’s wealth using the discounted cash flow model (DCF), which does not allow considering the market condition via the risk-neutral approach. The state value determinants are divided into two classes, determinants and sub-determinants. We presented a model to determine the optimal value of the marginal return on assets for making a governmental decision. Traditional DCF issues including pricing intangible components, cash flow uncertainty, and asset marginal yield jumps were taken into account.

## 1. Introduction

The traditional method for calculating the wealth of nations uses the Discounted Cash Flows (DCF) model to discount the cash flows of the country’s assets. Thus, the traditional DCF only considers the physical assets to calculate the wealth of nations [1–4]. In reality, there are many valuable assets other than the physical assets, such as the human capital and the reputation of the industry of the country. The reputation of the country makes the producers of this country ask for a premium on their products. Thus, the reputation factor can increase the GDP of the country and the wealth of this country at the same time. On the other hand, some financial components were not been priced in the previous studies, such as the net foreign assets. Ruback (2011) [5] considered uncertainty in cash flows by presenting adjusted cash follows formula to minimize this bias.

Sometimes DCF is not possible for intangible assets. DCF is criticized because it depends on future cash flows, that may be affected by uncertain events, while it is based on a historical data and certain assumptions [6]. In general, DCF has several problems in application. Many investigations interested in considering the problems of the DCF method for valuation and how could it be improved? For example, Reinert (2020) [7], investigated German Income Approach (GIA) valuation and DCF model and found that both of them could be a proxy for market prices. Polat and Battal (2021) [8] see that DCF doesn’t consider extraordinary scenarios and how the management reacts to it. They compared real option and DCF valuation methods and concluded that the real option valuation method is more optimistic than DCF. Espinoza et al. (2022) [9] criticized traditional valuation methods based on DCF. They concluded a difficulty in applying the DCF method to consider the climate-related risks in valuing the long-term assets. To consider this problem, they presented the decoupled net present value (DNPV) method. This model includes the risk and risk-reduction measures into the valuation process.

Fortunately, the World Bank report (2018) [10] considered human capital and the net foreign assets using the traditional method of evaluating assets. The traditional ways for pricing the assets use the Discounted Cash Flows (DCF) method. The DCF considers the discounted cash follows of the assets and sometimes considers the real probabilities of these cash follows to estimate the expected discounted cash follows of these assets. Unfortunately, the DCF does not price the external factors affecting the price of these assets; such as the inflation rate and the technology level. Furthermore, the DCF also does not enable us from considering the stochastic movements of the cash flows. Thus, DCF makes it difficult to evaluate the performance of the government and its ability to manage the returns and the risks of the country’s assets.

To solve this dilemma, we used the Ornstein-Uhlenbeck model. The Ornstein-Uhlenbeck model allows us to consider the dynamic nature of the return of any asset. Considering the movement of the return around a determined average is one of the advantages of the Ornstein-Uhlenbeck model. Using this model enables us to determine the coefficient of return reversion towards its mean. We can also estimate the speed of the asset mean reversion. Thus, we easily can predict the future price behaviour of the asset. So, using the Ornstein-Uhlenbeck model to evaluate the asset price gives more accurate results. To get more gains from using the Ornstein-Uhlenbeck model, we adjusted it so that we could consider the external factors that affect the asset’s return.

We included the coefficients of the non-priced factors into the Ornstein-Uhlenbeck model, which enable us to consider the effect of the external factors, such as the inflation rate and the technology level, in the pricing process. Hence, this paper aims to answer the following questions: First, how can we measure the state value? Second, how can we consider the determinants of the state value in the pricing models? Finally, what is the optimal marginal yield of assets to make a specific decision?

The contribution of this paper may be concluded as follows: calculate the state value considering the economic factors, estimate the risk of the state assets, and determine the optimal marginal yield of assets to make a specific decision.

The next parts will be divided into several sections. Section 2 reviews the state value sub-determinants and the perspectives of the state value. Then we presented the model in the third section. The fourth section shows the state value and a model to determine the optimal marginal yield of assets to make a specific decision. For clarification, we presented a numerical example in Section 4 also, then we presented a robustness test in the same section. Finaly, we discussed the concepts and the theoretical ideas in the paper and compared it with the literature.

## 2. Literature review

Many studies tried to find a determined value for the wealth of nations [11–13]. In the next sections, we will display these studies and the added value of the current study to their contributions.

### 2.1. The state value sub-determinants

Many researchers and organizations presented visions of the wealth of nations. For example, Adam Smith made a paradigm shift by thinking of labour as one of the nation’s assets.

Simon Kuznets defined the nation’s wealth as the stock of country wealth [11]. Shirras (1949) advised each country to estimate its national wealth every five years at least. Shirras see that the wealth of nations should not include the natural’s free gifts. Such as waterfalls, fish in the sea, and climate. However, in reality, the economic benefits from the gifts of nature. Thus, in the current paper, we did not follow Shirras’s approach to calculate the state value. We consider any asset the country can benefit from. Thus, we considered fisheries and forests’ rents.

Michael Porter introduced new types of national wealth by introducing “The Competitive Advantage of Nations”, that the nation has much wealth increasing its competitiveness, such as education and health, etc. [12]. Moreover, Credit Suisse S.A. each year publishes the Global Wealth Report Data Book, which includes the net wealth of countries. The book includes the real estate prices, exchange rates, equity market prices, liabilities, the adult population, human resources, natural resources, and capital and technological advancements.

In valuing the country’s assets, most papers take into account only three factors as influence factors that lead the asset prices. For example, Schwartz (1997) [13] used the three-factor model to price the future contracts of the commodity in order to value financial and real assets. These factors are the logarithm of the spot price of the commodity, the convenience yield of the commodity, and the interest rate.

Hamilton (2003) estimated the wealth of about 100 countries. He used the traditional DCF method to estimate the assets’ prices. Concluded that a large number of countries, particularly low-income countries, get an important share of their wealth from natural resources. Hamilton illustrated the importance of applying the portfolio approach to public investments. The advantage of our study is that it prevents a dynamic model to estimate the price and risk of the state’s assets. Thus, it may present an accurate resource to apply the portfolio approach in allocating country investments.

Mohun (2005) investigated the methodology of Shaikh and Tonak (Measuring the Wealth of Nations, 1994) and concluded that this method is unreliable and using better approximations is reasonable.

This paper will call these factors the “determinants” of the state value. In reality, these factors themselves are affected by other factors such as the inflation rate, the country’s reputation, the governance level, the economic geography, and the infrastructure. We will call these factors the “sub-determinants” of the state value. The contribution of this article is considering the sub-determinants in the pricing model.

In the next section, we will display extra factors, sub-determinants, which may affect the state value, considering that the main determinants will be covered within the model. In this paper, the sensitivity of the three factors of the Schwartz (1997) [13] to the previous sub-determinants will be considered by the coefficient $\beta_{i}$. The following part will study the sub-determinants of the state value:

#### 2.1.1. The country’s reputation.

The reputation refers to the aggregated evaluation of past, present, and future actions of a company, which makes an organization credible and trustworthy [14]. Therefore, the reputation of a country depends on people’s experiences and countries’ views, which in turn determine the behaviour patterns, attitudes, and activities of people and countries concerning a given country.

In the dynamic, contemporary, knowledge-based, technology economy era, one of the most important assets of a country’s wealth is its reputation [15].

In this paper, we not only consider the reputation of businesses or other organizations or individuals, but also of countries.

The country’s reputation guarantees achieving strategic advantage and continuing development prospects. This advantage will oblige states to try to preserve their international reputation. Whereas, the countries that violate international law will have a poor reputation, which leads other states to avoid cooperative opportunities with them in the future, reducing their competitiveness position on the global market, and reducing investment. Poor reputation also leads to escaping talented workers, tourists, consumers, scientists, artists, etc. Therefore, one of the most valuable merits, which helps the country build its wealth, is its reputation. Consequently, countries put in every effort to facilitate a positive perception of their country by maintaining its merits and values for an excellent reputation [16].

Countries can create their good reputation through the quality of their brands, the efficiency of their internal and external policies, the efficiency of attracting foreign investment and entrepreneurs, the efficiency of attracting skilled workers, the efficiency of attracting scientists and students. and others, their culture and national heritage, the view of their citizens whether they are famous or unknown, and the size of their tourism [17].

Li and Martin (2016) [18] illustrated a positive relationship between entrepreneurs reputation and funding formation outcomes.

Anokhin et al. (2021) [19] considered the impact of the reputation of corporate venture capital firms on their ability to attract investments. The study found a positive effect of a reputation for experience, active participation in the business, and misconduct on the ability of a corporate venture capital firm to attract investments.

Akyildirim et al. (2024) [20] investigated the effect of several types of reputational events on the financial performance of airlines considering the economic cycles. They illustrated that the effect of reputational events on the financial performance of airlines varies depending on the economic cycle. Their study concluded a sharp negative effect of reputational events during economic crises.

The Reputation Institute has been measuring countries’ reputations since 1999. Indicators of countries’ reputation are calculated based on 16 attributes within three areas: Effective government: safe place, ethical country, responsible participant in the global community, progressive social and economic policies, operates efficiently, favourable environment for business, appealing environment: friendly and welcoming, beautiful country, appealing lifestyle, enjoyable country, advanced economy: contributor to global culture, high-quality products & services, well-educated and reliable workforce, well-known brands, values education, technologically advanced. So, we can say that reputation depends on many factors, such as natural resources, geography, political system, culture, traditions, and customs [21].

Because of the importance of the country’s reputation, several investigations are interested in it. Fernandez-Crehuet et al. (2019) [22] created an index to measure the reputation of a country based on a financial markets perspective to see how attractive a country is for foreign investors by neutral, constant, and official measurement criteria. The index is a combination of four different dimensions: (1) markets and trade, (2) national accounts, (3) labour market, (4) living conditions and security, along with 23 distinct factors. A country’s international reputation index could be used to assist public policies designed to improve reputation in countries where it is needed. The study and analysis of this index will make it possible to measure the evolution of the factors that affect the country, both positively and negatively, in order to identify both strengths and weaknesses.

Dimitrova et al. (2017) [23] investigate the correlation between the export volume to the nation of origin and the reputation of the bilateral partner. The authors discover that each improvement in a world ranking of a nation’s reputation for products (in a target country) is related to a 2 percent rise in exports to that particular country; the impact is comparable to the importing country cutting a tariff by as much as 2.9 percent. Moreover, the authors discover that certain facets of a nation’s reputation—both for its people and its goods—attenuate different types of uncertainty and, as a result, promote export volume in various ways.

Some researchers are interested in the relationship between the reputation of the country and its institutions and the performance of the tourism sector. Albaladejo et al. (2015) [24] presented a dynamic econometric model of tourism demand in which the reputation impact is not constant but varies with congestion. Carrasco-Santos et al. (2021) [25] analyzed Marbella’s reputation on the Internet as a tourist destination and identification files according to social and demographic characteristics. The results show that Marbella is a luxurious shopping destination. The feature of this research paper is to deepen the Marbella reputation on the Internet by analyzing the specific attraction assessments.

On the other hand, some studies consider the effect of reputation.

In this paper, we will consider the country’s reputation in pricing the country’s assets using the time series of the Country Reptrak index. We selected this index because it is a popular index that anyone can get. This index also transforms the reputation as a qualitative variable to be a quantitative variable.

#### 2.1.2. Economic geography.

The economic geography refers to the connection between geography, location, and economics. In this paper, we will investigate how the location and geography affect the country’s economy. This leads to the question, “Does the economic geography consider one of the country’s wealth sub-determinants?

A country’s location and geography may directly affect sustainable economic development of the state through their effect on agricultural productivity, disease spared, the availability of natural resources (see, [26–28]), and institutional quality [26,29]. The location and geography may also reduce the country’s transport costs [30,31].

The geography determines the country’s ability to reach the global markets, which in turn has a positive effect on the country’s level of income.

In the last decades, the new economic geography has gradually gained interest from authors. One of the topics that has attracted the attention of researchers is the migration of talents between economic regions.

Wang et al. (2018) [32] consider the spatial positioning’s effects on the birth of new ventures in a region and the death of existing firms during the course of an industry. The study concluded that an industry’s spatial distribution is important, as regional competitiveness is influenced by both the industry’s local agglomeration externalities and those of neighbouring clusters. Moreover, the results conducted on the manufacturing of telecommunication equipment in Canada show that proximity to strong agglomeration externalities in other locations boosts a place’s capacity to start more new businesses when an industry expands but reduces its capacity to support current businesses and launch more new ones when an industry shakes out.

Gu et al. (2024) [33] investigated the economic geography of talent movements between regions in China. They suggest that amenities and economic variables have an impact on the movement and redistribution of talent. Furthermore, the economic consequences are more important in the movement and redistribution of talent than the amenity benefits are.

Some studies are interested in the role of economic geography in digital economics. For example, Li et al. (2024) [34] assessed 110 cities in China’s Yangtze River Economic Belt based on their digital economy indices. The findings indicated a declining westward trend in the digital economy of the YREB. There was a clear distribution of the digital economy’s development throughout the YREB. The growth of the digital economy exhibited a strong positive spatial link. Furthermore, the cities’ development of the digital economy was primarily distributed according to a low-low agglomeration pattern.

Klie and Madlener (2024) [35] considered the feasibility of geographic concentration versus geographic diversification of onshore wind parks in Germany. Klie and Madlener found that geographic diversification enhances the economics of wind only slightly.

Zheng et al. (2024) [36] studied the effect of the regional economic integration strategy of China on the circular economy. Findings of the study illustrated the significant impact of the regional economic integration strategy on the circular economy and the positive geographical spillover benefits on nearby cities.

Yue et al. (2024) [37] considered the effect of financial geographic structure on the quality and variability of corporate innovation and argued that an increasing number of bank branches close to businesses enhances the number of inventions and green technology patents.

According to Fujita et al.(1999) [38], the economic geography may be measured using two measures; the distance from core economies (continuous variable) and if the country is landlocked or not (dummy variable). In this study, we will consider the economic geography as a continuous variable, while we will let the economic geography be a dummy variable for future research to simplify the idea.

#### 2.1.3. Economic and social infrastructure.

Infrastructure generally refers to the fundamental services and facilities that support a range of economic endeavors and thereby aid in the nation’s economic development, including banking and finance, education, health, transportation and communication, irrigation, energy, science, and machinery, among others [39,40]. It encompasses roads, trains, highways, airports, mass transportation, telecommunication via landlines, cell phones, and internet systems, sanitation, education, health, energy, gas, water supply, waste treatment, prisons, police, fire, and courts.

Some have even gone so far as to include green infrastructure, which attempts to improve quality of life and support a green economy by enhancing a variety of essential products and ecosystem services, such as clean water and air, food, and green spaces in metropolitan areas. A properly developed and implemented green infrastructure plan will go a long way in ensuring sustainable cities in the nation. Green infrastructure has been identified as one of the key approaches to mitigate the environmental impacts of urbanization, industrialization, climate change, and other environmental degradation [41].

Many academic works (see: [42–49]) discuss the significance of effective infrastructure in fostering sustainable economic development, both in the short and long term. particularly in developing countries [50].

There are several main reasons why infrastructure is important, as it improves global value chain participation. For example, Ketu and Wirajing (2024) [51] showed how infrastructure development enhances African global value chain participation; infrastructure enhances the green transformation of the urban economy (Qin et al., 2024) [52]; the green infrastructure may be used to enhance air quality by the deposition of airborne particulate matter [53]. Digital infrastructure could be useful to improve the environmental quality [54]. In developing countries, transport and communication technology infrastructure improves human development [55].

In this study, we will represent the infrastructure using the method of Calderón and Servén (2004). There are three indexes for the infrastructure stock. The first index is the telecommunications sector index, which is calculated with the number of main telephone lines per 1,000 workers. The second index is the power sector index. We can represent the infrastructure stock of the power sector by the electricity generating capacity of the economy, or the MW per 1,000 workers. The last index is the transportation sector, which may be represented by the length of the road network. The infrastructure may also be measured by the World Economic Forum index in the global competitiveness report. It is measured by three pillars (transport infrastructure, utility infrastructure, and ICT adoption) [56].

#### 2.1.4. The governance.

The governance introduces a good relationship between people and government, which includes efficiency, honesty, responsiveness, and quality [57]. Moreover, it refers to the capacity of the government to effectively formulate and apply sound policies, and earns the respect of the residents and the states for the institutions that govern economic and social activities [58]. And it’s an alternative to traditional tools of governing [59,60].

Good governance affects all countries’ aspects in many ways. First, it affects the quality of services provided to the citizens, and the quality of government work [61]. Second, it relates positively to the levels of economic growth [62–64]. Third, governance improved regulatory quality that can lead to promoting economic growth by creating more incentives for the private sector, helping the poor by creating more opportunities for entrepreneurship. Fourth, by the rule of law, judicial independence promotes a stable investment environment that leads to higher levels of investment and growth and thus helps in reducing poverty through efficient legal systems [62,65]. Fifth, international donors, such as international organizations like the IMF and the World Bank and countries; are using the governance to determine the eligibility of states for grants and aids [61,66,67].

The governance level could be measured via several indicators, such as The Worldwide Governance Indicators (WGIs).

Méndez-Picazo et al. (2012) [68] investigate the connection between economic growth, entrepreneurship, and governance by conducting an empirical analysis using the cases of 11 developed countries. According to Martı’nb and Ribeiro-Sorianoc, there is a positive correlation between economic growth and four WGI indicators, namely voice and accountability, government effectiveness, rule of law, and control of corruption.

Fayissa and Nsiah (2013) [69] investigate how the six characteristics of WGIs affect economic growth. Results show that governance has a different role in economic growth depending on income levels. They discover that 39 sub-Saharan African nations differ from one another due to effective governance.

Huang and Ho (2016) [70] examine the Granger causality between governance and economic growth in twelve Asian countries from 1996–2014. They classified the sample of the study as “Free”, “Partly Free”, and “Not Free” countries. The empirical findings demonstrate that, with the exception of South Korea, “Free” nations did not significantly demonstrate a causal relationship between economic development and most aspects of governance. Except for Thailand and Indonesia, “Partly Free” nations see economic progress as a result of Granger’s rule of law. Regarding “Not Free” nations, there is a strong causal relationship between economic growth and most aspects of governance, particularly with regard to the rule of law and government efficacy.

Bah et al. (2021) [71] examine the impact of governance quality on exports in 45 sub-Saharan African countries, finding that total and service exports are positively influenced by these indicators, except for government effectiveness.

Mahran (2023) [72] explores the significant impact of governance on economic growth in 116 countries, revealing that a 1% increase in governance boosts average growth by 1%, influencing neighboring nations.

The governance level could be measured using several indicators, such as The Worldwide Governance Indicators (WGIs).

#### 2.1.5. The efficiency of Markets (capital – goods & services – housing).

Efficient markets should pursue good governance rules, whether they are capital, workforce, goods, and services, and housing markets that help it promote the growth and development of the economy.

We will discuss the efficiency of the market (capital – goods & services – housing) in the next part:

Goods and services markets: The efficiency of goods and services markets may reduce transaction costs, encourage industrialization, increase the employment rate, promote aggregate demand and consumption, stimulate the growth of complementary industries, raise the saving rate and productivity, and create the investment climate. Therefore, the state should focus on increasing the market efficiency, because the improvement in market-enhancing governance conditions would promote growth in the advanced countries [73].

Using the Structure-Conduct-Performance paradigm, Kroupová et al. (2022) [74] examine the profitability of food processing enterprises in the Czech Republic. It concludes that technological efficiency has a larger marginal effect and that market power has no effect on performance.

The influence of product market regulation on innovation and the digital economy is examined by Litina et al. (2021) [75], which concludes that more regulation causes innovative activities to decrease. It also looks at how innovation is impacted by digital legislation.

With an emphasis on less developed nations, Zhang and Graham (2020) [76] examine the causal link between air travel and economic performance. It discovers a reciprocal causal association between aeroplane enplanement and employment in the service industry. The study explored several common flaws and inefficiencies in aviation markets in light of the basic connections between air travel and economic growth.

Financial market: its development will lead to economic development and growth. The financial sector system is standing behind in developing the developed countries more than the developing countries [77,78]. Some studies concluded that better industrialization is the basic factor to increase the stock market growth and enhance the economic growth of the economy [79–82].

Ngo and Le (2019) [83] examine the link between capital market development and banking efficiency in 86 different countries from 2006 to 2011. According to research, greater capital markets have less efficient banking systems, and global banking systems remain inefficient. It is advised to prioritize improving banking performance above developing the capital markets.

Adabor (2023) [84] looks at how financial development affects the rent from natural gas resources in developing nations such as Ghana. The findings indicate that resource extraction is encouraged by an effective financial system and that credit from the monetary sector and net domestic credit are important factors in this process. This implies that policymakers should take financial development into account when formulating policies.

The housing market: is a fundamental component of development. The construction industry has a positive effect on macroeconomics for several reasons. First, the housing sector plays an essential role in rising consumption, where the buying and selling of houses is a significant portion of the total value in an economy [85]. The second reason is that the housing sector plays a pivotal role in the growth of complementary industries, where several sectors of the economy benefit from increased housing. The most beneficiaries would be the construction and home renovation industries and the durable goods firms. The efficient housing market has multiplier effects on real estate agents, surveyors, notaries, and bankers; this has multiplier effects throughout the economy. Third, the efficient housing market creates profitable investment opportunities that attract foreign direct investment (FDI). Fourth, the housing markets increase the savings rate because it is one of the most important tools for “forced savings”. This is very useful because the saving represents future consumption, so it is an essential component of the economy. Fifth, because of the above, the housing sector is a motivation to increase the growth rate; this is because the efficient housing market increases the growth rate of small and medium businesses, labor abilities, and technology [86].

In this paper, the efficiency of the capital market could be measured by the market index (WEF, 2019) [56]. The efficiency of the product market is measured by the WEF index (WEF, 2019) [56]. The efficiency of the housing market can be measured by the Housing Market Index (HMI).

#### 2.1.6. Institutions and democracy.

Although the importance of geography and cultural to economic growth, the efficient of institutions are essential in explaining long-run economic growth [87].

More so than the environment, institutional efficiency may be a key factor in economic growth [88–93].

Three different streams of information were identified in the literature regarding the relationship between institutions and economic growth:

- The first is the rule of government effectiveness, regulatory quality, law, control of corruption, protection of property rights, contract enforcement, and economic freedom, for improving long-run economic performance [94–99]. those which mobilize savings for investment, and those that hold officials accountable by the public [100,101].
- The second is political and civil liberties, absence of violence, democracy, political stability, etc. [102–106].
- The third is sustainable development [107,108], which includes investments in technology, human and physical capital, production organization, and resource allocation [108,109]. It additionally includes the supply of technological and organizational factors [72], research and development (R&D) [96,108,110–113], and the development of resource distribution in the nation [108].

Therefore, long-term economic growth cannot be explained without considering the effectiveness of institutions [79,108].

The institution variable could be measured by the World Economic Forum’s index in the global competitiveness report. It is measured by eight pillars (security, social capital, checks and balances, public- sector performance, transparency, property rights, corporate governance, future orientation of government) [56].

On the other hand, Economic growth is significantly influenced by political freedom [106,107]. There are several reasons supporting the relationship between democracy and effective governance.

Democracy gives citizens the ability to peacefully and frequently replace corrupt and ineffective government regimes with ones that are more successful and efficient. It is possible that democratic regime leaders would choose to pay attention to the demands of the populace and endure criticism for winning their support in elections. People are unable to manage their governments and comprehend these hazards in the absence of a democracy, open political institutions, and a free press. Thus, over time, democracy improves the standard of government.

The democracy has increased educational coverage and positively impacted spending on education [114]. According to a wealth of evidence, democracies outspend autocracies on education and invest more in the development of human capital than do dictatorships. This difference can be attributed to pressure from interest groups and elections [115]. According to Stasavage (2005) [116], basic education is improved by democracy. As democracy grows, so does human capital [117–119]. Democracies, on the other hand, spend more on public basic and secondary education. These investments can have a significant return because even modest increases in education funding can have a significant impact on the welfare of the populace [96].

According to a number of studies (e.g., [120–126], democracy promotes beneficial technological progress and, thus, increases productivity.

Some discovered that they have a mutual relationship between them. The democratization is an antecedent process (cause) to technical and economic transformation (result). In particular, the major finding is that democratization is a driving force for technological change: more democratic countries, measured with liberal, participative, and constitutional democracy indices, have a greater degree of technology than less free and more authoritarian ones. In reality, “democracy richness” creates a faster rate of technological innovation with productive repercussions for the wellbeing and wealth of nations. These findings and projections lead to the conclusion that policymakers need to be mindful of beneficial links between democracy and technological innovation paths in order to promote the current economic growth and future technical progress of countries [127].

According to Baum and Lake (2003) [119], this implies that democratic institutions could be crucial in producing long-term gains in human welfare.

Some studies, such as Baum and Lake (2003) [119], used the popular index six-step Guttman scale of openness of executive recruitment to measure democracy. The index puts sex scales for democracy. Thus, the measurement of the democracy needs to use dummy variables, which are out of the interest of this paper. Hence, we let the democracy factor for the future research.

#### 2.1.7. Exchange rate.

The value of a nation’s currency in another nation’s currency is referred to as the foreign exchange [128]. Exchange rates can have a significant impact on how resources are allocated and local prices are set [129].

Numerous variables affect it, including economic growth, the rate of inflation, the rate of unemployment, the balance of payments deficit and surplus, etc. If the government borrowed money and ran a budget deficit to fund economic expansion, this will negatively impact future currency values unless it is handled carefully. On the other hand, finance through more exports and investment will influence the exchange rate positively.

A nation’s economy can be impacted by exchange rates either favorably or unfavorably [130]. It might have an impact on the state’s capacity to achieve GDP growth and sustainable development [131–133]. As a result, it takes into account a significant component that influences a nation’s susceptibility to crises.

On the other hand, it was discovered by Hausmann, R., Pritchett, L., and D. Rodrik (2005) [134] that there is frequently a correlation between real exchange rate depreciation and strong growth accelerations. Relapsing international competitiveness and employment losses could have detrimental implications on trade and economic growth. Aman, Q., Ullah, I., Khan, M.I., and Khan, S. (2013) [135] demonstrated how an increase in exports and an improvement in investment might result from an exchange rate shift that is favorable to economic growth. According to Glüzmann, P. A., Levy-Yeyati, E., and F. Sturzenegger (2012) [136], the exchange rate increases domestic savings and investment as well as employment in developing nations by reducing labor costs and income redistribution. It has no effect on the trade sector.

However, there are restrictions on the exchange rate drop because moderate undervaluation promotes growth while significant overvaluation hinders it [137,138].

The exchange rate can be measured by the Exchange Rate Index.

### 2.2. The perspectives of state value

The traditional approach for evaluation of any asset is the Discounted Cash Flow (DCF). However, the DCF method does not consider the stability of the cash DCF inputs [6,139], such as the cash follows, the risk attached with these cash follows, and the factors that affect cash follows. The cash flow of any asset is affected by many factors, such as technological (the technology) progress, the inflation rate, and ESG (Environmental, Social, and Governance) [140]. Unfortunately, traditional DCF fail to consider the influence of uncertainty of cash flows on asset value [139]. The DCF may consider the inflation and the growth rate by multiplying the discounting part of the DCF formula by the inflation rate or the growth rate. The DCF does not consider the stochastic movement of the inputs. To illustrate this point, let’s assume that we price a mine. If we use the DCF model for capital budgeting, the mine value will be affected by the changes in the copper price. The DCF model does not consider the shocks in the copper price or the shock in the discounting rate. In other words, the DCF model for capital budgeting failed to consider uncertainty in the cash flows of the projects. This opinion was empirically illustrated by Ruback (2011) [5].

Several attempts were made to solve this problem [1–4]. However, all of them treated intangible assets as one number with showing the effect of each component of it. For example, if we calculate the value and the cost intangible assets according to Schauten et al. (2010) [4], we can know the effect of each factor affect intangible assets. For more explanation, assume that intangible assets are determined by several variables, such as human capital and governance, what is the effect of each factor on intangible assets? In this paper, we solve this problem by using a separate coefficient for each factor affects intangible assets and including these factors into the valuation model.

Intangible assets are not publicly traded (Schauten et al., 2010) [4], this make a restriction on using CAPM to calculate the cost of intangible assets. The model of this study solve this dilemma by replacing the traditional beta with the beta coefficient of the marginal return of each asset of the country against each factor affect the cash follows of this asset including intangible assets and tangible assets.

The evolution of capital budgeting’s algorithms produced the future model, which is one of the most popular models nowadays, especially in the oil companies. The future contract approach gives the decision maker the flexibility in timing market and choosing optimal time to do anything. According to the future contract model, the price of the underlying asset follows a geometric Brownian motion (GBM) process. This flexibility is not available in the NBV model.

#### 2.2.1. Evaluating the state assets on the micro-level.

Many researchers attempted to price the assets of the state as separate units. These studies priced stand-alone projects. For example, they considered estimating the fair value of mines and oil futures contracts. The oil futures models are suitable to both the future contracts and the real option projects.

The contribution of Brennan and Schwartz (1985) [141] is important on the micro-level. Brennan and Schwartz (1985) [141] evaluated the assets when the cash flows determined by future management decisions, not by the current cash flows. Brennan and Schwartz (1985) [141] did not present a comprehensive look for astablesets of the country. They evaluated only the mining and other natural resource projects. The evaluation of the state value requires pricing all components of the wealth, such as produced capital and urban land, natural capital, human capital, and net foreign assets (The Changing Wealth of Nations, 2018) [134].

Cortazar and Schwartz (1997) [141,142] presented an evaluation model for undeveloped oil fields in case of no arbitrage. The researchers continued in presenting a richer model to price the real projects.

Schwartz (1997) [13] developed two and three factor models for commodity prices. The models are suited to financial and real option pricing.

Cortazar, G., Schwartz, E. S., & Casassus, J. (2003) [143] considered a three-factor model to price oil futures contracts. The model can be used in other future contracts and in asset pricing.

Zhang and Sun (2018) [144] estimated the ocean wealth of China. Their study calculated the ocean wealth by summing three types of wealth, which are: ocean manufactured capital, ocean human capital, and ocean natural capital. Beside the previous components, they considered the two factors affecting the ocean wealth. These factors are the effect of environmental externalities and the population change. Zhang and Sun presented the value of the ocean wealth without investigating the volatility and the stochastic movement of the components of the wealth. The presented model depended only on constant parameters. In this paper, we treated this issue by assuming that the assets satisfy a Brownian motion.

Although the previous models were effective in evaluating the assets of the country and making decisions on it, they are not qualified to represent the performance of the government at all. The earlier models miss the comprehensive look for the country.

We concluded the problems of the micro-level approach:

- The micro-level approach only takes a snapshot of the assets of the country. Such as mines, copper, and other natural resource projects. The micro-level approach is also interested in pricing the foreign assets and the intangible assets. According to this approach, the assets of market value are separately calculated for each asset. Using this approach, we can evaluate the performance only of the management of this asset. The problem now is that the micro-level approach does not give us a comprehensive look for the entire asset of the country. Thus, we cannot evaluate the performance of the government when we use the micro-level approach.
- The micro-level approach does not give the government the ability to make decisions regarding the total assets of the country, such as changing the interest rate and imposing an extra tax.
- Although some studies in the micro-level approach considered the real option approach in evaluating the country’s assets, they did not include the effect of many factors on the assets. For example, Brennan and Schwartz (1985) [141] did not consider the effect of the technology shocks on the evaluated assets.

#### 2.2.2. Evaluating the state value using the macro-level approach.

These studies considered a comprehensive look for the assets of the country. The World Bank (2018) [10] presented four components for evaluating the total wealth of the country. These components are natural capital, produced capital, human capital, and net foreign assets. The natural capital includes minerals, agricultural land, comprising energy, protected areas, and forests.

Although the World Bank report presented a comprehensive look for the position of the country’s wealth, the report mainly depended on the DCF method. The report assumed that the assets’ values are stationary. The report did not consider the stochastic nature of the asset prices and the cash flows. The report may help the reader know the value of the nation’s wealth, but it may not help the decision maker in this country to know the optimal time to a special decision. The World Bank report does not enable us to consider any other factor that affects the assets. For example, the education level could not be considered in this model when we price the state assets. The traditional way of evaluating the assets of the country does not allow for considering the market effect.

In this paper, we solve these problems by using the future contract model, which enables the user of the model to achieve optimality. We used the Ornstein-Uhlenbeck process, which enables us to price other factors in the model. We presented a new contribution by considering the strength of the relationship between the asset price and the priced factors. The strength here refers to the regression coefficient between the asset price and the priced factors. In this paper, we call the strength coefficient a contribution because the Ornstein-Uhlenbeck process is not considering this coefficient.

## 3. The model

In order to calculate the value of the state and determine the optimal value of the marginal return on assets at which a certain economic decision is accepted by the government, we will take several steps. The first step involves identifying the factors that affect the value of the state’s assets and the sensitivity of the prices of those assets to these factors.

The second step is to calculate the value of the state by entering the previous factors into the fair value calculation model of the state, which takes into account the expected return on each of the state’s assets in addition to the factors affecting these returns.

The third step involves calculating the optimal value of the marginal return on assets at which a certain economic decision of the state is accepted or rejected.

According to the World Bank report (2018) [10], the state value includes natural capital, produced capital, human capital, and net foreign assets. We will evaluate each component separately using our model, considering the future contract approach:

### 3.1. The natural capital

The natural capital includes many components, such as the agriculture assets, the energy and mineral resources, and the protected areas and forests. We will price each asset in the following sections.

#### 3.1.1. The agriculture assets.

Assume that the agriculture asset price follows the next geometrical Brownian motion.


dS(t)=μS(t)dt+ηS(t)dW(t) (1)

$$d\;ar(s)=k\beta_{1}(Ar-ar)ds+\sigma dZ(s)$$ (2)

where μ is the drift of the asset price model, η represents the volatility of assets, and dW(t) is the increment to the Brownian motion of the asset price.

The price of an asset is affected by the shocks in agricultural expansions. The agricultural expansions satisfy the Ornstein-Uhlenbeck process, as the equation (2) reflects.

In equation (2), k is the speed of mean reversion of the agricultural expansions return ar to its long-run mean Ar. ar and Ar represent the instantaneous marginal yield of agricultural expansions, and long-run mean, respectively; so ar reverts to Ar.  dZ(s) reflects the increment to the Brownian motion of the agricultural expansions.

In reality, the time series of the marginal return on assets is not stationary, and it has jumps that affect its expected value exactly as other variables may affect its value.

The effect of jumps in the economic factors time series has a wide interest in the last decades. While some studies consider the jumps in returns of financial assets (e.g., [140]).

Ball and Torous (1983) [140] proposed a simplified model to consider the jump process for common stock returns. They found that the abnormal returns of stocks are due to received information which is expressed as a Poisson process.

Chan and Maheu (2002) [145] examined jump dynamics in equity market returns using conditional jump model. They concluded that the conditional jump intensity and jump size are varying over time.

Yeh and Yun (2023) [146] considered co-jumps and co-volatility between financial assets. They illustrated that both co-jumps and co-volatility cause extreme returns (jumps) and extreme dependence in time series of assets.

Movahed and Noshad (2024) [147] investigated the possibility of treating any changes in data time series as a jump, whatever it is, in a normal time or in a jump time. They called this process jump‑jump modelling.

In this paper, we consider the effect of a jump in the time series of the marginal yield of assets as follows:

Assume that the marginal yield of the state asset satisfies the following process:


$$ar=\beta_{10}+\beta_{11}ecf_{1}+\ldots +\beta_{1i}ecf_{1i}+\xi J+$$

where $\beta_{10}$ is the intercept of the regression, $\beta_{1i}$ is the regression coefficient of the instantaneous marginal yield of agricultural expansions towards the economic factor $ecf_{1i}$. ξ is the is the jump size and J is the jump arrival satisfying a Bernoulli distribution. Thus, J takes the value of 0 or 1. The strength of the current equation comes from considering both the sensitivity of the marginal yield of agricultural expands toward economic factors and the abnormal movement of the sensitivity of the marginal yield of agricultural expands at the same time.

The contribution of this paper is $\beta_{1}$, which represents the sensitivity coefficient of the agricultural expansions toward the external factors 1,2,3,…,i (sub-determinants), such as the inflation rate, exchange rate, etc. We can estimate the $\beta_{1}$ coefficient via the following equation:


$$\beta_{1}=\;\beta_{11}\times \beta_{12}\times \beta_{13}\times \ldots \times \beta_{1i}$$

where i represents any important external factor that affects the agricultural expansions, such as the inflation rate, exchange rate, etc. The external factor may include the reputation, which could be represented by the indicators such as the Reputation Institute Index. (In the appendix (5), we consider calculating the sensitivity of assets to economic geography factor).

Including $\beta_{1}$ in the model changes the state of the ar variable from an exogenous variable to an endogenous variable, so its value is estimated outside the model. We think the pricing model will be more realistic after adding $\beta_{1}$. We can integral equation (2) as:


$$\int_{t}^{T}d\;ar(s)=\int_{t}^{T}k\beta_{1}(Ar-ar)ds+\int_{t}^{T}\sigma dZ(s)$$ (3)

The previous equation may be rewritten as follows:


$$\int_{t}^{T}d\;ar(s)=k\beta_{1}Ar(T-t)-k\beta_{1}\int_{t}^{T}ar(s)d\;s+\sigma \int_{t}^{T}d\;Z(s)$$

So the cumulative yield of agricultural expansions from the date t until T is:


$$ar(T)-ar(t)=\;\int_{t}^{T}ar(s)$$ (4)

As in Bjerksund (1991) [148], we assumed that cumulative agricultural expansions yield could be expressed as follows:


$$X(t)\equiv \int_{0}^{T}ar(s)ds\;$$

where X(t) is the agricultural expansions cumulative return for the period from 0 to t.

The agricultural expands cumulative return, for the period from 0 to T, X(T) may be calculated by inserting X(t) into equation (4) results in (1 in S1 Appendix).


$$X(T)-X(t)\equiv \int_{t}^{T}ar(s)ds$$ (5)

Substituting for equations (4), (5) in equation (3), we get


$$ar(T)-ar(t)=\;k\beta_{1}Ar(T-t)-k\beta_{1}\left(X(T)-\;X(t)\right)+\;\sigma \int_{t}^{T}d\;Z(s)$$ (6)

The equation (6) gives us the agricultural expansions cumulative return for the period from t to T. Now it is easy to drive the asset value, as we will see.

**3.1.1.1.Evaluating the asset value and the variance considering the agricultural expansions:** We may write the initial formula for evaluating the fair value of the asset S on the date T (we do this evaluation on the date t):


$$V\left[S(T)\right]=e^{-r(T-t)}E^{*}\left[S(T)\right]$$

According to Bjerksund (1991) [148], the discounted future value of the asset can be expressed as follows:


$$e^{-r(T-t)}S(T)=S(texp\left\{\left(\mu -r+ar-\frac{1}{2}\eta^{2}\right)(T-t)\right\}+\eta \int_{t}^{T}d\;W(s)$$ (7)

In Appendix (1), we derived the integral of the Brownian motion $\int_{t}^{T}dw(s$ of the asset price as:


$$\int_{t}^{T}dw(s)=\;\int_{t}^{T}dw^{*}(s)-\;\frac{\mu -r}{\eta \;}\;(t-t)-\;\frac{1}{\eta \;}\;\left(X(T)-X(t)\right)$$ (8)

We can get the value of *X*(*T*) from the extraction of Appendix (3)

Thus,


η (9)

Multiplying the equation (8) by η and using the equation (9) (from Appendix (3)) to substitute for X(T)−X(t

This results in


$$\eta \int_{t}^{T}d\;W(s)=\eta \left[\int_{t}^{T}d\;W^{*}(s)\right]-\frac{\mu -r}{\eta }(T-t)-\frac{1}{\eta }\left(X(T)-\;X(t)\right)$$

$$\int_{t}^{T}d\;W(s)=\eta \int_{t}^{T}d\;W^{*}(s)-(\mu -r)(T-t)-\left(X(T)-\;X(t)\right)$$

Where we get the value (X(T)− X(t)) of the equation (9) and we substitute it in the previous equation, and thus we get:


$$\begin{array}{c} \begin{array}{c} \int_{t}^{T}d\;W(s)=\eta \int_{t}^{T}d\;W^{*}(s)-(\mu -r)(T-t)-\;Ar(T-t)-\frac{1}{k\beta_{1}}\sigma \int_{t}^{T}d\;Z(s)\\ +\frac{1}{k\beta_{1}}e^{-k\beta_{1}(T-t)}ar(t)-\frac{1}{k\beta_{1}}Ar\left(1-e^{-k\beta_{1}(T-t)}\right)+\frac{\sigma }{k\beta_{1}}e^{-k\beta_{1}T}\int_{t}^{T}e^{k\beta_{1}s}d\;Z(s)-\frac{ar(t)}{k\beta_{1}} \end{array} \end{array}$$ (10)

Inserting the equation (10) in the equation (7), we get the future discounted pay-off.


$$\begin{array}{cc} e^{-r(T-t)}S(T) & =S(t)\exp\{\left(\mu -r+ar-\frac{1}{2}\eta^{2}\right)(T-t)+\eta \int_{t}^{T}d\;W^{*}(s)-(\mu -r)(T-t)-\;Ar(T-t)-\frac{1}{k\beta_{1}}\sigma \int_{t}^{T}d\;z(s)\\ & \;+\frac{1}{k\beta_{1}}e^{-k\beta_{1}(T-t)}ar(t)-\frac{1}{k\beta_{1}}Ar\left(1-e^{-k\beta_{1}(T-t)}\right)+\frac{\sigma }{k\beta_{1}}e^{-k\beta_{1}T}\int_{t}^{T}e^{k\beta_{1}s}d\;Z(s)-\frac{ar(t)}{k\beta_{1}}\} \end{array}$$ (11)

Rearranging the previous equation, we get


$$\begin{array}{cc} e^{-r(T-t)}S(T) & =S(t)\exp\{(T-t)\left(\mu -r+ar-\frac{1}{2}\eta^{2}-\mu +r-Ar\right)+\eta \int_{t}^{T}d\;W^{*}(s)-\frac{1}{k\beta_{1}}\sigma \int_{t}^{T}d\;Z(s)\\ & \;+\frac{1}{k\beta_{1}}\left(e^{-k\beta_{1}(T-t)}ar(t)-Ar\left(1-e^{-k\beta_{1}(T-t)}\right)-ar(t)\right)+\frac{\sigma }{k\beta_{1}}e^{-k\beta_{1}T}\int_{t}^{T}e^{k\beta_{1}s}d\;Z(s)\}\\ & =S(t)\exp\{(T-t)\left(ar-\frac{1}{2}\eta^{2}-Ar\right)+\frac{1}{k\beta_{1}}\left(e^{-k\beta_{1}(T-t)}ar(t)-Ar\left(1-e^{-k\beta_{1}(T-t)}\right)-ar(t)\right)\\ & \;+\eta \int_{t}^{T}d\;W^{*}(s)-\frac{1}{k\beta_{1}}\left(\sigma \int_{t}^{T}d\;Z(s)-\sigma e^{-k\beta_{1}T}\int_{t}^{T}e^{k\beta_{1}s}d\;Z(s)\right)\} \end{array}$$

From equation (8), we can easily get the value of $\sigma \int_{t}^{T}d\;z(s)$. Thus, the future discounted pay-off may be written as (2 in S1 Appendix):


$$\begin{array}{cc} e^{-r(T-t)}S(T) & =S(t)\exp\{(T-t)\left(ar-\frac{1}{2}\eta^{2}-Ar\right)+\frac{1}{k\beta_{1}}\left(e^{-k\beta_{1}(T-t)}ar(t)-Ar\left(1-e^{-k\beta_{1}(T-t)}\right)-ar(t)\right)+\eta \int_{t}^{T}d\;W^{*}(s)\\ & \;-\frac{1}{k\beta_{1}}\left(\sigma \int_{t}^{T}d\;Z^{*}(s)-\sigma \int_{t}^{T}\lambda_{1}d(s)-\sigma e^{-k\beta_{1}T}\int_{t}^{T}e^{k\beta_{1}s}d\;Z^{*}(s)+\sigma e^{-k\beta_{1}T}\int_{t}^{T}e^{k\beta_{1}s}\lambda_{1}d(s)\right)\} \end{array}$$ (11B)

where $\lambda_{1}$ is the market price per unit risk of agricultural return.

Thus, the expected value of this asset (agriculture, agricultural sector) can be calculated as follows:


$$E=S(t)\exp\left\{(T-t)\left(ar-\frac{1}{2}\eta^{2}-Ar\right)+\frac{1}{k\beta_{1}}\left(e^{-k\beta_{1}(T-t)}ar(t)-Ar\left(1-e^{-k\beta_{1}(T-t)}\right)-ar(t)\right)\right\}$$

On the other hand, the variance is,


$$var=\hat{\sigma^{2}}\equiv E_{t}^{*}\left[{\left(Z^{*}\right)}^{2}\right]-{\left(E_{t}^{*}\left[Z^{*}\right]\right)}^{2}$$

where


$${\left(E_{t}^{*}\left[Z^{*}\right]\right)}^{2}=\mu^{2}$$

The previous magnitude represents the average of the X square.

$E_{t}^{*}\left[{\left(Z^{*}\right)}^{2}\right]$ (The first part of the variance equation) represents the mean of the square of $Z^{*}$.

Where μ is obtained from the equation (11) when we remove from it the variance.


$$E_{t}^{*}\left[{\left(Z^{*}\right)}^{2}\right]=E_{t}^{*}\left[{\left(\eta \int_{t}^{T}d\;W^{*}(s)-\frac{1}{k\beta_{1}}\sigma \int_{t}^{T}d\;Z^{*}(s)-\frac{1}{k\beta_{1}}\sigma e^{-k\beta_{1}T}\int_{t}^{T}e^{k\beta_{1}s}d\;Z^{*}(s)\right)}^{2}\right]$$

We can easily decompose each part of the previous equation as follows:

-
$\;{\left[\eta \int_{t}^{T}d\;W^{*}(s)\right]}^{2}=\eta^{2}(T-t)$

-
${\left[\frac{1}{k\beta_{1}}\sigma \int_{t}^{T}d\;Z^{*}(s)\right]}^{2}={\left(\frac{1}{k\beta_{1}}\right)}^{2}\sigma^{2}(T-t)$

-
${\left[\frac{1}{k\beta_{1}}\sigma e^{-k\beta_{1}T}\int_{t}^{T}e^{k\beta_{1}s}d\;Z^{*}(s)\right]}^{2}={\left(\frac{1}{k\beta_{1}}\right)}^{2}\frac{\sigma^{2}}{2k\beta_{1}}\left(1-(\theta {)}^{2}\right)={\left(\frac{1}{k\beta_{1}}\right)}^{i2}\frac{\sigma^{2}}{2k\beta_{1}}\left(1-{\left(e^{-k\beta_{1}(T-t)}\right)}^{2}\right)={\left(\frac{1}{k\beta_{1}}\right)}^{2}\frac{\sigma^{2}}{2k\beta_{1}}\left(1-e^{-2k\beta_{1}(T-t)}\right)$

• $\left[\left(\eta \int_{t}^{T}d\;W^{*}(s)\mathrm{\backslash rightleft}(\frac{1}{k\beta_{1}}\sigma \int_{t}^{T}d\;Z^{*}(s)\right)\right]=\frac{1}{k\beta_{1}}\sigma \eta \rho (T-t)$
• $\left[\left(\eta \int_{t}^{T}d\;W^{*}(s)\mathrm{\backslash rightleft}(\frac{1}{k\beta_{1}}\sigma e^{-k\beta_{1}T}\int_{t}^{T}e^{k\beta_{1}s}d\;Z^{*}(s)\right)\right]={\left(\frac{1}{k\beta_{1}}\right)}^{2}\sigma \eta \rho (1-\theta )={\left(\frac{1}{k\beta_{1}}\right)}^{2}\sigma \eta \rho \left(1-e^{-k\beta_{1}(T-t)}\right)$
• $\left[\left(\frac{1}{k\beta_{1}}\sigma \int_{t}^{T}d\;Z^{*}(s)\mathrm{\backslash rightleft}(\frac{1}{k\beta_{1}}\sigma e^{-k\beta_{1}T}\int_{t}^{T}e^{k\beta_{1}s}d\;Z^{*}(s)\right)\right]={\left(\frac{1}{k\beta_{1}}\right)}^{3}\sigma^{2}\left(1-e^{-k\beta_{1}(T-t)}\right)$

Inserting the previous parts into the equation of $E_{t}^{*}\left[{\left(Z^{*}\right)}^{2}\right]$ gives


$$\begin{array}{cc} E_{t}^{*}\left[{\left(Z^{*}\right)}^{2}\right] & =[\eta^{2}(T-t)-\frac{1}{k\beta_{1}}\sigma \eta \rho (T-t)-{\left(\frac{1}{k\beta_{1}}\right)}^{2}\sigma \eta \rho \left(1-e^{-k\beta_{1}(T-t)}\right)-\frac{1}{k\beta_{1}}\sigma \eta \rho (T-t)\\ & \;+{\left(\frac{1}{k\beta_{1}}\right)}^{2}\sigma^{2}(T-t)+{\left(\frac{1}{k\beta_{1}}\right)}^{3}\sigma^{2}\left(1-e^{-k\beta_{1}(T-t)}\right)-{\left(\frac{1}{k\beta_{1}}\right)}^{2}\sigma \eta \rho \left(1-e^{-k\beta_{1}(T-t)}\right)\\ & \;+{\left(\frac{1}{k\beta_{1}}\right)}^{3}\sigma^{2}\left(1-e^{-k\beta_{1}(T-t)}\right)+{\left(\frac{1}{k\beta_{1}}\right)}^{2}\frac{\sigma^{2}}{2k\beta_{1}}\left(1-e^{-2k\beta_{1}(T-t)}\right)] \end{array}$$

In order to calculate the variance, the following values must be calculated:


$$var=\hat{\sigma^{2}}\equiv E_{t}^{*}\left[{\left(Z^{*}\right)}^{2}\right]-{\left(E_{t}^{*}\left[Z^{*}\right]\right)}^{2}$$

where the second part of the variance equation is


$$\begin{array}{cc} E_{t}^{*}\left[Z^{*}\right] & \equiv \hat{\mu }=(T-t)\left(ar-\frac{1}{2}\eta^{2}-Ar\right)+\frac{1}{k\beta_{1}}\left(e^{-k\beta_{1}(T-t)}ar(t)-Ar\left(1-e^{-k\beta_{1}(T-t)}\right)-ar(t)\right)\\ & \;-\frac{1}{k\beta_{1}}\left(-\sigma \int_{t}^{T}\lambda_{1}d(s)+\sigma e^{-k\beta_{1}T}\int_{t}^{T}e^{k\beta_{1}s}\lambda_{1}d(s)\right)\\ & =(T-t)\left(ar-\frac{1}{2}\eta^{2}-Ar\right)+\frac{1}{k\beta_{1}}(e^{-k\beta_{1}(T-t)}ar(t)-Ar\left(1-e^{-k\beta_{1}(T-t)}\right)-ar(t)\\ & \;+\sigma \int_{t}^{T}\lambda_{1}d(s)-\sigma e^{-k\beta_{1}T}\int_{t}^{T}e^{k\beta_{1}s}\lambda_{1}d(s)) \end{array}$$ (12)

Thus, variance can be calculated var or $\hat{\sigma^{2}}$ as follows:


$$var=\hat{\sigma^{2}}\equiv E_{t}^{*}\left[{\left(Z^{*}\right)}^{2}\right]-{\left(E_{t}^{*}\left[Z^{*}\right]\right)}^{2}$$

The value of the asset is:


$$V^{*}\left[S(T)\right]=E_{t}^{*}\left[e^{-k\beta_{1}(T-t)}S(T)\right]=E_{t}^{*}\left[S(t)\exp\left\{Z^{*}\right\}\right]=S(t)\exp\left\{\hat{\mu }+\frac{1}{2}\hat{\sigma^{2}}\right\}\;$$ (13)

**3.1.1.2.Evaluating the agriculture assets considering three factors:** In the previous discussion, we examined the agriculture assets, considering only the effect of the agricultural expansions on yields. We will now study the state value bearing in mind the technology effect.

Joseph Schumpeter (2002) [149] defines innovation as an activity that leads to new production.

In this section, we investigate the value of the agriculture assets considering agricultural expansions yields and Technological effect. The technological effect may be expressed through education level.

The agriculture asset price satisfies the next geometrical Brownian motion:


dS(t)=μS(t)dt+ηS(t)dW(t) (1)

$$d\;ar(s)=k\beta_{1}(Ar-ar)ds+\sigma dZ(s)$$ (2)

$$d\;edlev=\;\alpha \beta_{2}(EdLev-\;edlev)ds+\gamma dh\;(s)$$ (14)

where

edlev represents the instantaneous marginal yield of technological effect.
EdLev reflects the long-run mean of the technological effect yield and which the EdLev reverts to it, α is the speed of mean reversion of the technological effect return edlev to its long-run mean EdLev.
γdh (s) reflects the increment to the Brownian motion of the technological effect.
$\beta_{2}$ represents the sensitivity coefficient of the technological effect toward the external factors 1,2,3,…,i (sub-determinants). As we showed in the two factors pricing model, the $\beta_{2}$ coefficient may be calculated by:

$$\beta_{2}=\;\beta_{21}\times \beta_{22}\times \beta_{23}\times \ldots \times \beta_{2i}$$

The price of an asset is affected by the shocks in agricultural expansions and the technological effect. The technological effect return satisfies the Ornstein-Uhlenbeck process, as the equation (14) showed.

The integral of the instantaneous marginal yield of the technological effect equation may be obtained by getting the integral of equation (14) represented as follows:



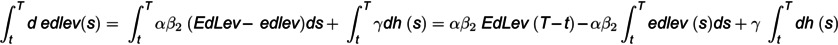

(15)


From the previous equation, we could easily extract the following equations:


$$Edlev(T)-edlev(t)=\int_{t}^{T}d\;edlev(s)$$ (16)

$$Y(T)-Y(t)\equiv \int_{t}^{T}edlev(sds$$ (17)

$$edlev(T)-edlev(t)=\alpha \beta_{2}\;EdLev\;(T-t)-\alpha \beta_{2}\left(Y(T)-Y(t)\right)+\gamma \int_{t}^{T}dh(s)$$ (18)

It is easy now to calculate the asset value considering the considering agricultural expands yields and technological effect.


$${V\left[S(T)\right]=e}^{-r\;(T-t)}E^{*}\left[S(T)\right]$$ (19)

The previous equation represents the initial formula for evaluating the asset S on the date T. From Bjerksund (1991) [148], ***footnote* [4]**, the future value is:


$$e^{-r(T-t)}S(T)=S(t)exp\;\left\{\left(\mu -r+ar+edlev-\;\frac{1}{2}{\eta \;}^{2}\right)(T-t)+\;\eta \;\int_{t}^{T}dW(s)\right\}$$ (20)

Multiplying the $\int_{t}^{T}dw(s$value (3 in S1 Appendix) by the η and using the **Appendix 4** to substitute for X(T)−X(t, we get the value of $\eta \;\int_{t}^{T}dW(s$ in equation (21) as follows:


$$\eta \int_{t}^{T}dW(s)=\;\eta \;\left[\int_{t}^{T}dW^{*}(s)-\;\frac{\mu -r}{\eta \;\;}\;(T-t)-\;\frac{1}{\eta }\;\left(X(T)-X(t)\right)-\;\frac{1}{\eta \;\;}\;(Y(T)-Y(t)\right]$$

$$\eta \;\int_{t}^{T}dW(s)=\;\int_{t}^{T}dW^{*}(s)-(\mu -r)\;(T-t)-\;\left(X(T)-X(t)\right)-\;\left(Y(T)-Y(t)\right)$$

where (X(T)−X(t)) could be obtained from equation (9), (Y(T)−Y(t)) could be obtained from equation (6), **Appendix 4**. Substituting with the value of both (X(T)−X(t)) and (Y(T)−Y(t)) it in the previous equation, we get:


$$\begin{array}{cc} \eta \;\int_{t}^{T}dW(s) & =\int_{t}^{T}dW^{*}(s)-(\mu -r)(t-t)-\;Ar(T-t)-\;\frac{1}{k\beta_{1}}\sigma \;\int_{t}^{T}dZ(s)+\;\frac{1}{{k\beta }_{1}}e^{-{k\beta }_{1}\;(T-t)}\;ar(t)-\;\frac{1}{{k\beta }_{1}}\;Ar\;\left(1-e^{-k\beta_{1}(T-t)}\right)\\ & \;+\frac{\sigma }{k\beta_{1}}e^{-k\beta_{1}T}\int_{t}^{T}e^{k\beta_{1}s}dZ(s)-\frac{ar(t)}{{k\beta }_{1}}-EdLev\;(T-t)-\;\frac{1}{\alpha \beta_{2}}\gamma \int_{t}^{T}dh(s)+\;\frac{1}{{\alpha \beta }_{2}}e^{-{\alpha \beta }_{2}\;(T-t)}edlev(t)\\ & \;-\frac{1}{\alpha \beta_{2}}EdLev\;\left(1-\;e^{-{\alpha \beta }_{2}\;(T-t)}\right)+\;\frac{\gamma }{\alpha \beta_{2}}\;e^{-{\alpha \beta }_{2}T}\;\int_{t}^{T}e^{\alpha \beta_{2}S}dh(s)-\;\frac{edlev\;(t)}{\alpha \beta_{2}} \end{array}$$ (21)

Inserting the equation (21) in equation (20), we get the future discounted pay-off as follows


$$e^{-r(T-t)}s(T)=s(t)\exp\left\{\left(\mu -r+ar+edlev-\;\frac{1}{2}{\eta \;}^{2}\right)(T-t)+\;\eta \;\;\int_{t}^{T}{dW}^{*}\;(s)-(\mu -r)(T-t)-Ar\;(T-t)-\;\frac{1}{k\beta_{1}}\sigma \;\int_{t}^{T}dZ(s)+\;\frac{1}{{k\beta }_{1}}e^{-{k\beta }_{1}\;(T-t)}\;ar(t)-\;\frac{1}{{k\beta }_{1}}\;Ar\;\left(1-e^{-k\beta_{1}(T-t)}\right)+\frac{\sigma }{k\beta_{1}}e^{-k\beta_{1}T}\int_{t}^{T}\;\;e^{k\beta_{1}s}\;dZ(s)-\frac{ar(t)}{{k\beta }_{1}}-EdLev\;(T-t)-\;\frac{1}{\alpha \beta_{2}}\gamma \int_{t}^{T}dh(s)+\;\frac{1}{{\alpha \beta }_{2}}e^{-{\alpha \beta }_{2}\;(T-t)}edlev(t)-\;\frac{1}{\alpha \beta_{2}}EdLev\;\left(1-\;e^{-{\alpha \beta }_{2}\;(T-t)}\right)+\;\frac{\gamma }{\alpha \beta_{2}}\;e^{-{\alpha \beta }_{2}T}\;\int_{t}^{T}e^{\alpha \beta_{2}S}dh(s)-\;\frac{edlev\;(t)}{\alpha \beta_{2}}\right\}$$

Rearranging the previous equation,


$$\begin{array}{cc} e^{-r(T-t)}s(T) & =s(t)\exp\{\left(\mu -r+ar+edlev-\;\frac{1}{2}{\eta \;}^{2}-\;\mu +r-Ar-EdLev\right)(T-t)\\ & \;+\;\frac{1}{{k\beta }_{1}}\left(e^{-{k\beta }_{1}\;(T-t)}\;ar(t)-\;Ar\;\left(1-e^{-k\beta_{1}(T-t)}\right)-ar(t)\right)\\ & \;+\frac{1}{{\alpha \beta }_{2}}\left(e^{-{\alpha \beta }_{2}\;(T-t)}edlev(t)-\;EdLev\;\left(1-\;e^{-{\alpha \beta }_{2}\;(T-t)}\right)-edlev\;(t)\right)\\ & \;+\eta \;\;\int_{t}^{T}{dW}^{*}\;(s)-\frac{1}{k\beta_{1}}\left(\sigma \;\int_{t}^{T}dZ(s)-\sigma e^{-k\beta_{1}T}\int_{t}^{T}\;\;e^{k\beta_{1}s}\;dZ(s)\right)\\ & \;-\frac{1}{{\alpha \beta }_{2}}\left(\gamma \int_{t}^{T}dh(s)-\gamma e^{-{\alpha \beta }_{2}T}\;\int_{t}^{T}e^{\alpha \beta_{2}S}dh(s)\right)\} \end{array}$$

In other words, we can calculate the expected value of the asset by the next formula:


$$\begin{array}{cc} e^{-r(T-t)}S(T) & =S(t)\exp\{(T-t)\left(ar+edlev-\;\frac{1}{2}{\eta \;}^{2}-Ar-EdLev\right)+\;\frac{1}{{k\beta }_{1}}\left(e^{-{k\beta }_{1}\;(T-t)}\;ar(t)-\;Ar\;\left(1-e^{-k\beta_{1}(T-t)}\right)-ar(t)\right)\\ & \;+\;\frac{1}{{\alpha \beta }_{2}}\left(e^{-{\alpha \beta }_{2}\;(T-t)}edlev(t)-EdLev\left(1-e^{-{\alpha \beta }_{2}\;(T-t)}\right)-edlev(t)\right)\\ & \;+\eta \;\;\int_{t}^{T}{dW}^{*}\;(s)-\frac{1}{k\beta_{1}}(\sigma \;\int_{t}^{T}dZ^{*}(s)-\sigma \;\int_{t}^{T}\lambda_{1}d(s)\\ & \;-\sigma e^{-k\beta_{1}T}\int_{t}^{T}\;\;e^{k\beta_{1}s}\;dZ^{*}(s)+\sigma e^{-k\beta_{1}T}\int_{t}^{T}\;\;e^{k\beta_{1}s}\;d(s)))\\ & \;-\frac{1}{{\alpha \beta }_{2}}\left(\gamma \int_{t}^{T}dh^{*}(s)-\;\gamma \int_{t}^{T}\lambda_{2}d(s)-\gamma e^{-{\alpha \beta }_{2}T}\;\int_{t}^{T}e^{\alpha \beta_{2}S}dh^{*}(s)+\;\gamma e^{-{\alpha \beta }_{2}T}\;\int_{t}^{T}e^{\alpha \beta_{2}S}\lambda_{2}d(s)\;\right)\} \end{array}$$ (22)

where $\lambda_{1}$, $\lambda_{2}$ are the market price per unit risk of agricultural expands yields and technological effect.

In order to calculate variance, the following values must be calculated:


$$var={\hat{\sigma }}^{2}\equiv E_{t}^{*}\left[{\left(Z^{*}\right)}^{2}\right]-{\left(E_{t}^{*}\left[Z^{*}\right]\right)}^{2}$$

As we previously showed, μ is obtained from the modified equation (22) when we remove the variance from it. Thus, the first part of the variance equation is


$$\begin{array}{cc} E_{t}^{*}\left[{\left(Z^{*}\right)}^{2}\right] & =E^{*}[(\eta \;\int_{t}^{T}{dW}^{*}\;(s)-\;\;\frac{1}{k\beta_{1}}\sigma \;\int_{t}^{T}dZ^{*}(s)+\frac{1}{k\beta_{1}}{\sigma e}^{-k\beta_{1}T}\int_{t}^{T}\;\;e^{k\beta_{1}s}\;dZ^{*}(s)\\ & \;{-\;\frac{1}{\alpha \beta_{2}}\gamma \int_{t}^{T}dh^{*}(s)+\;\frac{1}{\alpha \beta_{2}}\gamma e^{-{\alpha \beta }_{2}T}\;\int_{t}^{T}e^{\alpha \beta_{2}S}dh^{*}(s))}^{2}] \end{array}$$

Using the same concept of the previous section, we extract the following values (we can solve each part of the previous equation as following):

-
$\left[\eta \;(\;\int_{t}^{T}{dW}^{*}\;(s)){]}^{2}=\;{\eta \;}^{2}\;(T-t\right)$

-
$\left[(\frac{1}{K\beta_{1}}\;\sigma \;\int_{t}^{T}dZ^{*}(s)){]}^{2}={\left(\frac{1}{K\beta_{1}}\right)}^{2}\;\sigma^{2}\;(T-t\right)$

-
${\left[\left(\frac{1}{\alpha \beta_{2}}\;\gamma \int_{t}^{T}dh^{*}(s)\right)\right]}^{2}={\left(\frac{1}{\alpha \beta_{2}}\right)}^{2}\;\gamma^{2}\;(T-t)$

-
${\left[\left(\frac{1}{k\beta_{1}}\;{\sigma e}^{-k\beta_{1}T}\int_{t}^{T}e^{k\beta_{1}S}dZ^{*}(s)\right)\right]}^{2}={\left(\frac{1}{K\beta_{1}}\right)}^{2}\;{\frac{\sigma }{2K}}^{2}\;\left(1-\theta_{2}^{2}\right)$

-
$\theta_{2}^{2}={\left(e^{-k\beta_{1}(T-t)}\right)}^{2}=\left(e^{-2k\beta_{1}(T-t)}\right)$

-
${\left[\left(\frac{1}{\alpha \beta_{2}}\;{\gamma e}^{-\alpha \beta_{2}T}\int_{t}^{T}e^{\alpha \beta_{2}S}dh^{*}(s)\right)\right]}^{2}={\left(\frac{1}{\alpha \beta_{2}}\right)}^{2}\;{\frac{\gamma }{2\alpha }}^{2}\left(1-\theta_{2}^{2}\right)$

-
$\theta_{2}^{2}={\left(e^{-\alpha \beta_{2}(T-t)}\right)}^{2}=\left(e^{-2\alpha \beta_{2}(T-t)}\right)$

-
$\left[\left(\eta \int_{t}^{T}{dW}^{*}(s)\mathrm{\backslash rightleft}(\frac{1}{k\beta_{1}}\;\sigma \;\int_{t}^{T}dZ^{*}(s)\right)\right]=\frac{1}{k\beta_{1}}\;\sigma {\eta \;\rho }_{12}(T-t)$

-
$\left[\left(\eta \int_{t}^{T}{dW}^{*}(s)\mathrm{\backslash rightleft}(\frac{1}{k\beta_{1}}\;\sigma \;e^{-k\beta_{1}T}\int_{t}^{T}e^{k\beta_{1}s}dZ^{*}(s)\right)\right]={\left(\frac{1}{k\beta_{1}}\right)}^{2}\sigma {\eta \;\rho }_{12}\left(1-e^{-k\beta_{1}(T-t)}\right)$

-
$\left[\left(\eta \int_{t}^{T}{dW}^{*}(s)\mathrm{\backslash rightleft}(\frac{1}{\alpha \beta_{2}}\;{\gamma e}^{-\alpha \beta_{2}T}\int_{t}^{T}e^{\alpha \beta_{2}S}dh^{*}(s)\right)\right]={\left(\frac{1}{\alpha \beta_{2}}\right)}^{2}\gamma {\eta \;\rho }_{13}\left(1-e^{-\alpha \beta_{2}(T-t)}\right)$

-
$\left[\left(\eta \int_{t}^{T}{dW}^{*}(s)\mathrm{\backslash rightleft}(\frac{1}{\alpha \beta_{2}}\;\gamma \int_{t}^{T}dh^{*}(s)\right)\right]=\frac{1}{\alpha \beta_{2}}\gamma \;{\eta \;\rho }_{13}(T-t)$

-
$\left[\left(\frac{1}{k\beta_{1}}\;\sigma \;\int_{t}^{T}dZ^{*}(s)\mathrm{\backslash rightleft}(\frac{1}{k\beta_{1}}\;\sigma \;e^{-k\beta_{1}T}\int_{t}^{T}e^{k\beta_{1}s}dZ^{*}(s)\right)\right]={\left(\frac{1}{k\beta_{1}}\right)}^{3}\sigma^{2}\left(1-e^{-k\beta_{1}(T-t)}\right)$

-
$\left[\left(\frac{1}{k\beta_{1}}\;\sigma \;\int_{t}^{T}dZ^{*}(s)\mathrm{\backslash rightleft}(\frac{1}{\alpha \beta_{2}}\;\gamma \int_{t}^{T}dh^{*}(s)\right)\right]=\left(\frac{1}{k\beta_{1}}\right)\left(\frac{1}{\alpha \beta_{2}}\right)\sigma \gamma \;\rho_{23}(T-t)$

-
$\left[\left(\frac{1}{k\beta_{1}}\;\sigma \;\int_{t}^{T}dZ^{*}(s)\mathrm{\backslash rightleft}(\frac{1}{\alpha \beta_{2}}\;{\gamma e}^{-\alpha \beta_{2}T}\int_{t}^{T}e^{\alpha \beta_{2}S}dh^{*}(s)\right)\right]=\left(\frac{1}{k\beta_{1}}\right)\left(\frac{1}{\alpha \beta_{2}}\right)\sigma \gamma \;\rho_{23}\left(1-e^{-\alpha \beta_{2}(T-t)}\right)$

To see the illustration of the expected values: $\left[\left(\frac{1}{k\beta_{1}}\;\sigma \;e^{-k\beta_{1}T}\int_{t}^{T}e^{k\beta_{1}s}dZ^{*}(s)\mathrm{\backslash rightleft}(\frac{1}{\alpha \beta_{2}}\;\gamma \int_{t}^{T}dh^{*}(s)\right)\right]$, $\left[\left(\frac{1}{k\beta_{1}}{\sigma e}^{-k\beta_{1}T}\int_{t}^{T}e^{k\beta_{1}S}dZ^{*}(s)\mathrm{\backslash rightleft}(\frac{1}{\alpha \beta_{2}}\gamma e^{-\alpha \beta_{2}T}\int_{t}^{T}e^{\alpha \beta_{2}s}dh^{*}(s)\right)\right]$, and $\left[\left(\frac{1}{\alpha \beta_{2}}\;\gamma \int_{t}^{T}dh^{*}(s)\mathrm{\backslash rightleft}(\frac{1}{\alpha \beta_{2}}\gamma e^{-\alpha \beta_{2}T}\int_{t}^{T}e^{\alpha \beta_{2}s}dh^{*}(s)\right)\right]$, please see the **Appendix 2.**

In order to calculate the variance, the following values must be calculated:

The first part of the variance equation:


$$\begin{array}{cc} E_{t}^{*}\left[{(z}^{*}{)}^{2}\right] & =[\eta^{2}(T-t)-\frac{1}{k\beta_{1}}\sigma \eta \rho_{12}\;(T-t)+\;{\left(\frac{1}{k\beta_{1}}\right)}^{2}\sigma \eta \rho_{12}\;\left(1-e^{-k\beta_{1}(T-t)}\right)\\ & \;-\;\frac{1}{\alpha \beta_{2}}\gamma \eta \rho_{13}\;(T-t)+{\left(\frac{1}{\alpha \beta_{2}}\right)}^{2}\gamma \eta \rho_{13}(1-e^{-\alpha \beta_{2}(T-t)})-\frac{1}{k\beta_{1}}\sigma \eta \rho_{12}\;(T-t)+{\left(\frac{1}{k\beta_{1}}\right)}^{2}\sigma^{2}\;(T-t)\\ & \;-\;{\left(\frac{1}{k\beta_{1}}\right)}^{3}\sigma^{2}\left(1-e^{-k\beta_{1}(T-t)}\right)+\left(\frac{1}{k\beta_{1}}\right)\left(\frac{1}{\alpha \beta_{2}}\right)\;\sigma \gamma \rho_{23}(T-t)\\ & \;-\;\left(\frac{1}{k\beta_{1}}\right)\left(\frac{1}{\alpha \beta_{2}}\right)\;\sigma \gamma \rho_{23}\;\left(1-e^{-\alpha \beta_{2}(T-t)}\right)+{\left(\frac{1}{k\beta_{1}}\right)}^{2}\sigma \eta \rho_{12}\left(1-e^{-k\beta_{1}(T-t)}\right)\\ & \;-{\left(\frac{1}{k\beta_{1}}\right)}^{3}\sigma^{2}\left(1-e^{-k\beta_{1}(T-t)}\right)+({\frac{1}{k\beta_{1}})}^{2}\;\frac{\sigma^{2}}{2k}\left(1-e^{-2k\beta_{1}(T-t)}\right)-\left(\frac{1}{k\beta_{1}}\right)\left(\frac{1}{\alpha \beta_{2}}\right)\sigma \gamma \rho_{23}\left(1-e^{-k\beta_{1}(T-t)}\right)\\ & \;+\;\left(\frac{1}{k\beta_{1}}\right)\left(\frac{1}{\alpha \beta_{2}}\right)\sigma \gamma \rho_{23}\left(1-e^{-k\beta_{1}(T-t)}\right)\left(1-e^{-\alpha \beta_{2}(T-t)}\right)\\ & \;-\;\frac{1}{\alpha \beta_{2}}\gamma \eta \rho_{13}\;(T-t)+\;\left(\frac{1}{k\beta_{1}}\right)\left(\frac{1}{\alpha \beta_{2}}\right)\sigma \gamma \rho_{23}(T-t)+\;\left(\frac{1}{k\beta_{1}}\right)\left(\frac{1}{\alpha \beta_{2}}\right)\sigma \gamma \rho_{23}\left(1-e^{-k\beta_{1}(T-t)}\right)\\ & \;+\;{\left(\frac{1}{\alpha \beta_{2}}\right)}^{2}\gamma^{2}(T-t)+{\left(\frac{1}{\alpha \beta_{2}}\right)}^{3}\gamma^{2}\left(1-e^{-\alpha \beta_{2}(T-t)}\right)+{\left(\frac{1}{\alpha \beta_{2}}\right)}^{2}\gamma \eta \rho_{13}\left(1-e^{-\alpha \beta_{2}(T-t)}\right)\\ & \;-\;\left(\frac{1}{k\beta_{1}}\right)\left(\frac{1}{\alpha \beta_{2}}\right)\sigma \gamma \rho_{23}\left(1-e^{-\alpha \beta_{2}(T-t)}\right)+\;\;\left(\frac{1}{k\beta_{1}}\right)\left(\frac{1}{\alpha \beta_{2}}\right)\sigma \gamma \rho_{23}\left(1-e^{-k\beta_{1}(T-t)}\right)\;\left(1-e^{-\alpha \beta_{2}(T-t)}\right)\\ & \;-{\left(\frac{1}{\alpha \beta_{2}}\right)}^{3}\gamma^{2}\left(1-e^{-\alpha \beta_{2}(T-t)}\right)+{\left(\frac{1}{k\beta_{2}}\right)}^{2}\frac{\gamma^{2}}{2\alpha }\left(1-e^{-2\alpha \beta_{2}(T-t)}\right)] \end{array}$$

The second part of the variance equation:


$$\begin{array}{cc} E_{t}^{*}[Z^{*}] & \equiv \hat{\mu }\equiv (T-t)\left(ar\;+\;edlev-\;\frac{1}{2}{\eta \;}^{2}-\;Ar\;–\;EdLev\right)\frac{1}{k\beta_{1}}\left(e^{-k\beta_{1}(T-t)}ar(t)\right)-Ar\;\left(1-\;e^{-k\beta_{1}(T-t)}-ar(t)\right)\\ & \;+\;\frac{1}{\alpha \beta_{2}}\left(e^{-\alpha \beta_{2}(T-t)}edlev\;(t)-EdLev\;\left(1-\;e^{-\alpha \beta_{2}(T-t)}\right)-edlev\;(t)\right)\\ & \;-\frac{1}{k\beta_{1}}\;\left(-\;\sigma \int_{t}^{T}\lambda_{1}d(s)+\;\sigma e^{-k\beta_{1}T}\int_{t}^{T}e^{k\beta_{1}s}\lambda_{1}d(s)\right)-\;\frac{1}{\alpha \beta_{2}}(-\;\gamma \int_{t}^{T}\lambda_{2}d(s)+\;\gamma e^{-\alpha \beta_{2}T}\int_{t}^{T}e^{\alpha \beta_{2}s}\lambda_{2}d(s)) \end{array}$$ (23)

Thus, the variance value or ${\hat{\sigma }}^{2}$ can be calculated as follows:


$$var={\hat{\sigma }}^{2}\equiv E_{t}^{*}\left[{\left(Z^{*}\right)}^{2}\right]-{\left(E_{t}^{*}\left[Z^{*}\right]\right)}^{2}$$

The value of the asset V is:


$$\begin{array}{cc} V_{t}^{*}\left[s(T)\right] & =E_{t}^{*}\left[e^{-r(T-t)}s(T)\right]=E_{t}^{*}\left[s(t)\right]e\left\{z^{*}\right\}=s(t)\exp\left\{\hat{\mu }+\frac{1}{2}{\hat{\sigma }}^{2}\right\}\\ & =E_{t}^{*}\left[s(T)e\left\{\varepsilon^{*}\right\}\right]=S(t)\;exp\;\left\{\hat{\mu }+\;\frac{1}{2}{\hat{\sigma }}^{2}\right\} \end{array}$$ (24)

#### 3.1.2. Energy and mineral assets.

Evaluating the energy and mineral resources projects requires similar steps, such as in the agriculture assets evaluation. Here, the method such as in equations (1), (2), and (14) will be used. The symbols in the previous equations would be replaced to fit the evaluation of the energy and mineral assets.

arand Ar will represent the instantaneous marginal convenience yield of the energy and mineral resources projects and the long-run mean, respectively. Thus, the ar reverts to Ar. The symbol k will represents the speed of mean reversion of the convenience yield return ar to its long-run mean Ar. The symbol edlev represents the instantaneous marginal yield of technological effect, and  EdLev reflects the long-run mean of the technological effect yield, which the edLev reverts to. The symbol α reflects the speed of mean reversion of the technological effect return return edlev to its long-run mean EdLev.

$\beta_{1}$represents the sensitivity coefficient of the convenience yield toward the external factors 1,2,3,…,i such as the economic geography, governance, reputation, inflation,

The coefficient $\beta_{2}$ shows the sensitivity of the technological effect yield toward the external factors 1,2,3,…,i. We may include the governance, reputation, the public expensive on the education, etc. dZ(s) represents the increment to the Brownian motion of the convenience yield.

Note: The education reputation in this case may be measured via a weighted average of the scores of the educational institutions regarding any respected ranking, such as the QS ranking.

#### 3.1.3. Protected areas and forests.

The value of the forest resources may be calculated by the capitalized value of rents from timber and non-timber services. We can easily estimate the value of the protected areas by capitalizing the rents of these assets.

Since the rents of the forest resources and the protected areas satisfy a geometric Brownian motion, the value of these assets could be estimated using the equations (1), (2), and (14).

The value of the natural capital could be calculated by the summation of agricultural land, the energy and mineral assets value, the forest resources, and the protected areas. The total value of the natural capital may be represented by the symbol NC.

### 3.2. Produced capital

The second component of the state value is the produced capital. The World Bank defined the produced capital as the manufactured or built assets such as machinery, equipment, and physical structures.

In general, there are several methods for evaluating the produced capital. For example, the perpetual inventory method is the certified method by the Organization for Economic Co-operation and Development (OECD) countries. One of the popular methods for evaluating the produced capital is to obtain it directly from the data of the national accounts. Here, the popular formula of the GDP will be used:


$$\begin{array}{cc} GDP & =\;private\;consumption\;+\;gross\;investment\;+\;government\;investment\\ & \;+\;government\;spending\;+\;(exports\;–\;imports). \end{array}$$

Whatever the method used to calculate GDP, the GDP series will satisfy the Brownian motion equation, and we can simply use the equations (13), and (24) to calculate the produced capital. Here, GDP represents the cash flows of the assets. In our model, we can represent the produced capital by the character PC.

We can use the GDP equation to consider several economic activities. For example, if we want to consider the international trade in the model, you may insert it in the export and import activities.

### 3.3. The net foreign assets

According to the World Bank methodology for calculating the foreign assets value, it may be estimated by the net foreign assets value. The net foreign assets value is calculated by subtracting total foreign liabilities from total foreign assets. The foreign assets include equity, FDI, debt, derivatives, and Forex. In this paper, we can evaluate equity, FDI, debt, and derivatives using the equations (13), and (24). Regarding Forex, we only need to translate it to the national currency. We can reflect the foreign assets value with FA.

### 3.4. Human capital

Human resources must be developed continuously and managed wisely to achieve sustainable development.

The countries can accumulate their human capital by developing the educational system and increasing the quality of the health system. Hence, there is a relation between the nation’s capability to productively use physical capital and the level of human capital, as weak human capital could hinder economic development.

This highlights the importance of the accumulation of human capital as the engine for economic growth alongside the other wealth factors. The improving of the education system speeds economic growth and improves citizens’ standard of living through many channels: it increases efficiency and so the productivity and so salaries (Psacharopoulos and Patrinos, 2004) [150], enhances democracy (Barro, 1997) [151], creates a good environment for good governance, increases equality, and improve the health system (Aghion et al., 1999) [152].

In the World Bank report (2018) [10] this studies priced the human capital considering the real probabilities of employing and the discount rate. The pricing model in the equations (13), and (24) used a risk-neutral measure so that we only needed to extract the expected earnings of each employee category $H_{ae}$. Each category of employee has an expected earning H, years of schooling by e, and age a. Each expected earnings will satisfy the Brownian motion and the equations (13), and (24). We can use the marginal rate of experience years and the mean of the technological effect yield as pricing factors.

The coefficient $\beta_{1}$ represents the sensitivity coefficient of the marginal rate of experience years to the external factors 1,2,3,…,i such as unemployment, inflation rate, exchange rate…etc. the $\beta_{2}$ represents the sensitivity coefficient of the technological effect to the factors 1,2,3,…,i such as public expenses on educations, research and developments, etc.

The value of the human capital could be reflected by HC.

## 4. The state value

In the previous sections, we displayed the calculation method for the fair value of each part of the state value. We used the future contract approach to estimate the economic value of state. The future contract approach enables us from considering the market premiums-adjusted- discounted value of the expected value of country assets instead of the traditional evaluation methods which consider the real probabilities and the discounted value without considering the market premiums of the factors affects the value of these assets. We can get the total state value using the following equation:


SV=NV+PC+FA+HC (25)

The previous equation enables us from evaluating the performance of the government and its ability to maximizing the wealth of the nation. The decision making will be a simple future contract model (or simple optimality problem) and we can use the smooth pasting method to get the optimal decision point. We can find the optimization point by find the first derivative of the state value function regarding any component of the state value (such as the agricultural land if the decision relate to the agricultural land) and equate it with zero.

### 4.1. The optimal marginal yield of agricultural expands for a specific decision

In the previous sections, we extract the future value of the state assets. Now, we can easily use this formula to make governmental decisions. For example, assume that the government evaluating increasing the agricultural land, The optimal agricultural assets price is the price which make the derivative of the future value of the agricultural assets with respect to yield of agricultural expands ar equal to zero. We can find the optimality equation by deriving equation (11-B).

Let’s start with following formula


$$E=S(texp\left\{(T-t)\left(ar-\frac{1}{2}\eta^{2}-Ar\right)+\frac{1}{k\beta_{1}}\left(e^{-k\beta_{1}(T-t)}ar(t)-Ar\left(1-e^{-k\beta_{1}(T-t)}\right)-ar(t)\right)\right\}$$ (26)

The value of ar that satisfies the condition $\frac{dE}{dar}=0$ from the previous equation may be extracted as following:

The differentiation of E with respect to ar is:


$$\begin{array}{cc} \frac{dE}{dar} & =S(t)\exp\left\{(T-t)\left(ar-\frac{1}{2}\eta^{2}-Ar\right)+\frac{1}{k\beta_{1}}\left(e^{-k\beta_{1}(T-t)}ar(t)-Ar\left(1-e^{-k\beta_{1}(T-t)}\right)-ar(t)\right)\right\}.\frac{d}{dar}\\ & \;\times \left\{(T-t)\left(ar-\frac{1}{2}\eta^{2}-Ar\right)+\frac{1}{k\beta_{1}}\left(e^{-k\beta_{1}(T-t)}ar(t)-Ar\left(1-e^{-k\beta_{1}(T-t)}\right)-ar(t)\right)\right\} \end{array}$$ (27)

The previous equation equal zero if the first part S(texp{…} or the second part $\frac{d}{dar}\left\{\ldots \right\}$ equal zero.

The second part of the previous equation $\frac{d}{dar}\{\ldots \}$ represents the derivative of the expression inside the exponent. We may decompose it into two parts as follows:


$$\frac{d}{dar}\left\{(T-t)\left(ar-\frac{1}{2}\eta^{2}-Ar\right)\right\}=(T-t)$$

$$\frac{d}{dar}\;\left\{\frac{1}{k\beta_{1}}\left(e^{-k\beta_{1}(T-t)}ar(t)-Ar\left(1-e^{-k\beta_{1}(T-t)}\right)-ar(t)\right)\right\}=\frac{1}{k\beta_{1}}\left(e^{-k\beta_{1}(T-t)}-1\right)$$ (28)

Combining these results and set it to zero


$$\begin{array}{cc} \frac{dE}{dar} & =S(t)\exp\left\{(T-t)\left(ar-\frac{1}{2}\eta^{2}-Ar\right)+\frac{1}{k\beta_{1}}\left(e^{-k\beta_{1}(T-t)}ar(t)-Ar\left(1-e^{-k\beta_{1}(T-t)}\right)-ar(t)\right)\right\}\\ & \;\times .\left((T-t)+\frac{1}{k\beta_{1}}\left(e^{-k\beta_{1}(T-t)}-1\right)\right)=0 \end{array}$$

Since S(texp{…} can’t be zero, we focus on the part inside the parentheses:


$$\left((T-t)+\frac{1}{k\beta_{1}}\left(e^{-k\beta_{1}(T-t)}-1\right)\right)=0$$

Unfortunately, this part doesn’t contain ar. Thus, we cannot extract the optimal value of ar from this part. So, we should decompose the first part of equation (27) to find the optimal value of ar.

Thus,


$$(T-t)=-\frac{1}{k\beta_{1}}\left(e^{-k\beta_{1}(T-t)}-1\right)$$

Rearranging gives:


$$e^{-k\beta_{1}(T-t)}=1-(T-t)k\beta_{1}$$

Mathematically, S(texp{…} equal zero if S(t) or exp{…} equal zero. Since S(t) cannot be zero, we should find the value of ar that make exp{…} equal zero. Since $e^{Constant}=0$, we have to find the value of ar that makes the exponent equal zero. Thus, we should solve the following equation for ar:


$$(T-t)\left(ar-\frac{1}{2}\eta^{2}-Ar\right)+\frac{1}{k\beta_{1}}\left(e^{-k\beta_{1}(T-t)}ar(t)-Ar\left(1-e^{-k\beta_{1}(T-t)}\right)-ar(t)\right)=\;C$$

where C represents a Constant. Rearranging the previous equation gives:


$$(T-t)ar(t)+\frac{1}{k\beta_{1}}e^{-k\beta_{1}(T-t)}ar(t)-\frac{1}{k\beta_{1}}ar(t)=C+(T-t)\left(\frac{1}{2}\eta^{2}+Ar\right)+\frac{1}{k\beta_{1}}Ar\left(1-e^{-k\beta_{1}(T-t)}\right)$$

Factor ar from the left side:


$$ar(t)\left((T-t)+\frac{1}{k\beta_{1}}e^{-k\beta_{1}(T-t)}-\frac{1}{k\beta_{1}}\right)=C+(T-t)\left(\frac{1}{2}\eta^{2}+Ar\right)+\frac{1}{k\beta_{1}}Ar\left(1-e^{-k\beta_{1}(T-t)}\right)$$

We can write ar(t) follows:



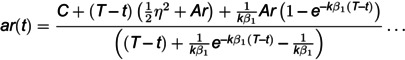

(29)


The previous equation simply shows the value of ar(t). However, determine the value of the constant C is a challenge. To simplify this dilemma, we will consider an initial value to ar(t) enables us to calculate C. We will use the long-run mean of marginal yield of agricultural expands. Since the long-run mean of marginal yield of agricultural expands equal Ar, as we assumed in Equation 2, the initial value of ar(t0) may be written as:


ar(t0)=Ar (30)

Substitute ar(t0) into the equation of C gives:



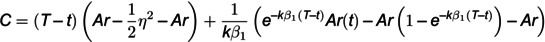

(31)


We may simplify each part of the previous equation a follows:

***First part***


$$\left(T-t)\left(Ar-\frac{1}{2}\eta^{2}-Ar\right)=(T-t)\left(-\frac{1}{2}\eta^{2}\right)=-\frac{1}{2}\eta^{2}(T-t\right)$$

***Second part***


$$\frac{1}{k\beta_{1}}\left(e^{-k\beta_{1}(T-t)}Ar(t)-Ar\left(1-e^{-k\beta_{1}(T-t)}\right)-Ar\right)=\frac{1}{k\beta_{1}}\left(2Are^{-k\beta_{1}(T-t)}-Ar\right)$$

Putting it all together


$$C=-\frac{1}{2}\eta^{2}(T-t)+\frac{1}{k\beta_{1}}\left(2Are^{-k\beta_{1}(T-t)}-Ar\right)$$ (32)

Now it is easy to estimate C and use it to find the optimal value of ar(t).

Inserting C into the equation of ar(t) gives:



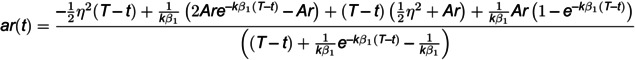

(33)


The previous equation may be simplified by the following steps:

The first part of the numerator


$$-\frac{1}{2}\eta^{2}(T-t)+(T-t)\left(\frac{1}{2}\eta^{2}+Ar\right)=Ar(T-t)$$

The second part of the numerator


$$\begin{array}{cc} & \frac{1}{k\beta_{1}}\left(2Are^{-k\beta_{1}(T-t)}-Ar\right)+Ar(T-t)+\frac{1}{k\beta_{1}}Ar\left(1-e^{-k\beta_{1}(T-t)}\right)\\ & \;=Ar\left((T-t)+\frac{1}{k\beta_{1}}\left(\left(2e^{-k\beta_{1}(T-t)}-1\right)+\left(1-e^{-k\beta_{1}(T-t)}\right)\right)\right)=Ar\left((T-t)+\frac{1}{k\beta_{1}}e^{-k\beta_{1}(T-t)}\right) \end{array}$$

The denominator


$$(T-t)+\frac{1}{k\beta_{1}}e^{-k\beta_{1}(T-t)}-\frac{1}{k\beta_{1}}=(T-t)+\frac{1}{k\beta_{1}}\left(e^{-k\beta_{1}(T-t)}-1\right)$$

Thus, the final expression of ar(t) is


$$ar(t)=\frac{Ar\left((T-t)+\frac{1}{k\beta_{1}}e^{-k\beta_{1}(T-t)}\right)}{\left((T-t)+\frac{1}{k\beta_{1}}\left(e^{-k\beta_{1}(T-t)}-1\right)\right)}$$ (34)

### 4.2. Numerical example

Let’s present a simple example to simplify our ideas. Assume that the Egyptian government evaluate an investment decision given that: T=10, t=5, Ar=3, k=0.2, $\beta_{1}=1.5\;$*.* Using the previous equation of calculating the optimal ar(t), the optimal ar(t)=5.80. This result shows that the government should not accept any instantaneous marginal yield of agricultural expands less than 5.80.

An important advantage of this model is that considers economic factors in making the decision. Another important advantage is that our model considers the stationarity of the economy by including k in the model. Since k represents the speed of mean reversion of the agricultural expands return ar(t) to its long-run mean Ar, The increase in the value of k means a high speed of mean reversion of the agricultural expands return ar(t) to its long-run mean Ar and a high level of stationarity. On the other hand, low level of k means low level of stationary.

### 4.3. Robustness test: Varying k

Now let’s examine the effect of varying speed of mean reversion of the agricultural expands return k on the optimal agricultural expands return ar(t). Let’s k and other terms are fixed. S1 Table shows that increasing k, the stability of marginal return, results in low level of the optimal marginal agricultural expands return. This is an expected results because increasing the level of stationarity reduce the degree of risk and make the government accept lower level of marginal return of agricultural expands.

## 5. Discussion and conclusion

This paper explores the concept of state value, providing a fresh perspective on the wealth of nations. We presented a mathematical formula to calculate the price of the state assets and the optimal marginal yield of the assets, which is accepted to make an economic decision. While the traditional DCF approach produces the discounted value of the state assets, our model considers the dynamic nature of the economy in the pricing process. We think this model differs from the competitiveness approach of Porter, who considers the competitive advantage of nations without presenting an obvious formula to calculate the state value. Although Schwartz (1997) [4] presented a model to price the mining assets, his model didn’t allow us to consider the assets of the country as one unit. In this paper, we presented a new insight to consider the state value by deriving models to calculate the state value and making decisions. Thus, we think the current model is an approximated insight into reality. We hope this model helps governments all over the world to evaluate their countries assets and evaluate their performance.

Although DCF has wide usage between analysts, it may have several disadvantages, such as difficulty in considering intangible assets. While some studies (Reilly and Schweihs, 1999 [1]; Mard et al., 2002 [2]; Smith and Parr, 2005 [3]; Schauten et al., 2010 [4] investigated this issue, it still has not the ability to decompose intangible assets to their original components, such as reputation, human capital, governance, etc. In this paper, we presented a model allows us to decompose the price of each component of the state value, including intangible assets. The problem of uncertainty of cash flows has a big share of the literature (Ruback, 2011 [5]; Šperanda, 2012 [6]; Reinert, 2020 [7]; Polat and Polat and Battal, 2021 [8]. However, too few studies are interested in the stochastic movement of cash flows. We used the Brownian motion process of the marginal return on states’ assets to solve this problem. Although some studies consider the Brownian motion of the marginal yield of assets (Schwartz,1997) [13], these investigations do not include the economic factors in the valuation model. This study contributes to literature by presenting a valuation model includes an unlimited number of factors affecting the marginal yield of states’ assets. The previous studies have considered DCF and uncertainty in cash flows. However, it didn’t investigate jumps in marginal yield of assets. In this paper, we contributed to the literature by including jumps of the marginal yield of assets in the state valuation model.

Fasen (2012) [153] investigated the multivariate Ornstein–Uhlenbeck model, focusing on the co-integration phenomenon between assets. The concept of this study is finding the pairs of co-integrated assets to consider them. Although her work is useful for several purposes, such as portfolio management and hedging funds, it is not qualified for our work. When we consider the state assets, it is not sure that all assets are co-integrated. For example, the forests’ rents are not sure co-integrated with the human capital assets. Thus, we may not consider the multivariate Ornstein-Uhlenbeck model in the current paper.

In this paper, we considered the state value as the value of any economic institution, which we can easily price its assets. We divided the determinants of the state value into two classes to evaluate them. The first is the determinants of state value which mentioned within the model. It included (natural capital, product capital, the net foreign assets, human capital). The second is the sub-determinants of the state value, which may have an impact on the first determinants. It included (country’s reputation, economic geography, economic and social infrastructure, governance, market efficiency, institutions, democracy, and exchange rate).

Evaluating the state assets enables us from making decisions by calculating the value before and after making any decision. Determining the value enables the decision maker from detecting the optimal time and the optimal value of the assets for any decision. We examined several cases of the state value. First, we modeled the pricing of the assets considering two factors, and then we include a third factor in the pricing model. The contribution of this paper includes also the possibility of include unlimited factors influencing the value of the assets. Although we used a long mathematical derivation, we ended our derivation with a simple formula to the state value and other formula for making decisions by the policy maker. Furthermore, we presented a new model to calculate the optimal value of the marginal return on assets which maximizes the marginal future value of the assets price in case of making decisions. We considered several problems belonging to traditional DCF, such as pricing intangible factors, uncertainty of cash flows, and jumps of the marginal yield of assets.

## Supporting information

S1 TableThe effect of varying speed of mean reversion of the agricultural expands return k on the *optimal* agricultural expands return ar(t).(XLSX)

S1 Appendix1) In equation (4) we use $\int_{t}^{T}ar(s)$, while in equation (5) we use $\int_{t}^{T}ar(s)ds$. 2) Assuming that $W^{*}=W-\overset{―}{W}$, $Z^{*}=Z-\overset{―}{Z}$, the correlation between the change of the two Brownian motions $W^{*}$ and $Z^{*}$ is $W^{*}\times Z^{*}=\rho$. 3) Using the same logic of deriving the equation (8) in the **Appendix (1)**, the $\int_{t}^{T}dw(s$ may be written as following: $\eta \;\int_{t}^{T}dw(s)=\;\int_{t}^{T}dw^{*}(s)-\;\frac{\mu -r}{\eta \;}\;(T-t)-\;\frac{1}{\eta \;}\;\left(X(T)-X(t)\right)-\;\frac{1}{\eta \;}\;(Y(T)-Y(tldots(8)$.(DOCX)
