# Supplementary material for: The state value
Source: PLoS One. 2025 Jun 17;20(6):e0320029. doi: 10.1371/journal.pone.0320029 (PMC12173242; doi:10.1371/journal.pone.0320029)
Supplement: S1 Appendix — 1) In equation (4) we use ∫tTar(s), while in equation (5) we use ∫tTar(s)ds. 2) Assuming that W*=W−W―, Z*=Z−Z―, the correlation between the change of the two Brownian motions W* and Z* is W*×Z*=ρ. 3) Using the same logic of deriving the equation (8) in the Appendix (1), the ∫tTdw(s may be written as following: η ∫tTdw(s)= ∫tTdw*(s)− μ−rη (T−t)− 1η (X(T)−X(t))− 1η (Y(T)−Y(tldots(8). (DOCX) [file pone.0320029.s002.docx]

**S1 Appendixes**

**Appendix (1)**

**The derivation of the integral of the assets price's Brownian motion** $\int_{\boldsymbol{t}}^{\boldsymbol{T}} \boldsymbol{dw}\left( \boldsymbol{s} \right)$

According to Gibson and Schwartz (1990) and Bjerksund (1991) [145], we can simply get $d W\left( s \right)$ by subtracting the risk premium for the incremental return on risk free return $\frac{\mu-r}{\eta} \left( T-t \right)$ from the expected value of the Martingale risk measure$\int_{t}^{T} dW^{*}\left( s \right)$. We need to consider the agricultural expansion factor in pricing the asset. Thus, we also will subtract risk premium for agricultural expansion $\frac{1}{\eta} \left( X\left( T \right)-X\left( t \right) \right)$ from$\int_{t}^{T} dW^{*}\left( s \right)$. We define the integral of the assets price's Brownian motion (The change in the price risk of the asset $\int_{t}^{T} dw\left( s \right)$) as following:

$$\int_{t}^{T} dw\left( s \right)=\int_{t}^{T} dw^{*}\left( s \right)- \frac{\mu-r}{\eta} \left( T-t \right)- \frac{1}{\eta} \left( X\left( T \right)-X\left( t \right) \right)\ldots\left( 8 \right)$$

We can get $\left( X\left( T \right)-X\left( t \right) \right)$ using equation $\left( 3 \right)$.

The value of the Martingale risk measure for the asset $S$ is $\int_{t}^{T} dW^{*}\left( s \right)$.

We subtracted the risk premium for additional asset over risk free return $\frac{\mu-r}{\eta} \left( T-t \right)$ from the Martingale risk measure $\int_{t}^{T} dW^{*}\left( s \right)$ then we subtracted the Agricultural expansion dividend risk premium $\frac{1}{\eta} \left( X\left( T \right)-X\left( t \right) \right)$.

**Appendix (2)**

- $E_{t}^{*}\left[ \left( \frac{1}{k\beta_{1}} \sigma e^{-k\beta_{1}T}\int_{t}^{T} e^{k\beta_{1}s}dZ^{*}\left( s \right) \right)\left( \frac{1}{\alpha\beta_{2}} \gamma\int_{t}^{T} dh^{*}\left( s \right) \right) \right]=\left( \frac{1}{k\beta_{1}}\sigma Z^{*}\left( s \right)(T-t)\left( 1-e^{-k\beta_{1}(T-t)} \right) \right)\left( \frac{1}{\alpha\beta_{2}}\gamma h^{*}\left( s \right)(T-t) \right)=\left( \frac{1}{k\beta_{1}} \right)\left( \frac{1}{\alpha\beta_{2}} \right)\sigma\gamma\rho_{23}\left( 1-e^{-k\beta_{1}\left( T-t \right)} \right)$
- $E_{t}^{*}\left[ \left( \frac{1}{k\beta_{1}}{\sigma e}^{-k\beta_{1}T}\int_{t}^{T} e^{k\beta_{1}S}dZ^{*}\left( s \right) \right)\left( \frac{1}{\alpha\beta_{2}}\gamma e^{-\alpha\beta_{2}T}\int_{t}^{T} e^{\alpha\beta_{2}s}dh^{*}\left( s \right) \right) \right]=\left( \frac{1}{k\beta_{1}} {\sigma Z}^{*}\left( s \right)(T-t)\left( 1-e^{-k\beta_{1}\left( T-t \right)} \right) \right)\left( \frac{1}{\alpha\beta_{2}}\gamma h^{*}\left( s \right)(T-t)\left( 1-e^{-\alpha\beta_{2}\left( T-t \right)} \right) \right)=\left( \frac{1}{k\beta_{1}} \right)\left( \frac{1}{\alpha\beta_{2}} \right)\sigma\gamma\rho_{23}\left( 1-e^{-k\beta_{1}\left( T-t \right)} \right)\left( 1-e^{-\alpha\beta_{2}\left( T-t \right)} \right)$

$$E_{t}^{*}\left[ \left( \frac{1}{\alpha\beta_{2}} \gamma\int_{t}^{T} dh^{*}\left( s \right) \right)\left( \frac{1}{\alpha\beta_{2}}\gamma e^{-\alpha\beta_{2}T}\int_{t}^{T} e^{\alpha\beta_{2}s}dh^{*}\left( s \right) \right) \right]=\left( \frac{1}{\alpha\beta_{2}} \right)^{3}\gamma^{2}\left( 1-e^{-\alpha\beta_{2}\left( T-t \right)} \right)$$

**Appendix (3)**

**Derivation of the agricultural expands cumulative return, for the period from 0 to T (**$\boldsymbol{X}\left( \boldsymbol{T} \right)$**) (equation** $\left( \boldsymbol{9} \right)$**):**

According to the equation $\left( 18 \right)$, the agricultural expands cumulative return for the period from $t$ to $T$ may be written as:

$$ar\left( T \right)-ar\left( t \right)= k\beta_{1}Ar\left( T-t \right)-k\beta_{1}\left( X\left( T \right)- X\left( t \right) \right)+ \sigma\int_{t}^{T} d Z\left( s \right)$$

By inserting the modified $ar\left( T \right)$^^[[1]](#footnote-1)^^ equation $\left( 5 \right)$ in the left side of the equation $\left( 18 \right)$ we can get the equation $\left( 6 \right)$ as follows:

$$e^{-{k\beta}_{1} \left( T-t \right)} ar\left( t \right)- \left( 1-e^{-k\beta_{1}\left( T-t \right)} \right)Ar+\sigma e^{-k\beta_{1}T}\int_{t}^{T} e^{k\beta_{1}s} dZ\left( s \right)-ar\left( t \right)=k\beta_{1}Ar\left( T-t \right)-k\beta_{1}\left( X\left( T \right)- X\left( t \right) \right)+ \sigma\int_{t}^{T} d Z\left( s \right)$$

Rearranging the previous equation, we get the value of $k\beta_{1}\left( X\left( T \right)- X\left( t \right) \right)$:

$$k\beta_{1}\left( X\left( T \right)- X\left( t \right) \right)=k\beta_{1}Ar\left( T-t \right)+ \sigma\int_{t}^{T} d Z\left( s \right)-e^{-{k\beta}_{1} \left( T-t \right)} ar\left( t \right)+ \left( 1-e^{-k\beta_{1}\left( T-t \right)} \right)Ar-\sigma e^{-k\beta_{1}T}\int_{t}^{T} e^{k\beta_{1}s} dZ\left( s \right)+ar\left( t \right)$$

Dividing both sides by $k\beta_{1}$ and rearranging, we can calculate $X\left( T \right)$:

$$X\left( T \right)= X\left( t \right)Ar\left( T-t \right)+ \frac{1}{k\beta_{1}}\sigma\int_{t}^{T} d Z\left( s \right)-\frac{1}{k\beta_{1}}e^{-{k\beta}_{1} \left( T-t \right)} ar\left( t \right)+\frac{1}{k\beta_{1}}Ar\left( 1-e^{-k\beta_{1}\left( T-t \right)} \right)-\frac{\sigma}{k\beta_{1}}e^{-k\beta_{1}T}\int_{t}^{T} e^{k\beta_{1}s} dZ\left( s \right)+\frac{ar\left( t \right)}{k\beta_{1}}\ldots\left( 9 \right)$$

**Appendix (4)**

**Deriving the agricultural expands cumulative return and the technological effect yield, for the period from 0 to T (**$\boldsymbol{X}\left( \boldsymbol{T} \right)$**) (equation** $\left( \boldsymbol{6} \right)$**):**

Remember the equation $\left( 18 \right)$

$$edlev\left( T \right)-edlev\left( t \right)=\alpha\beta_{2} EdLev \left( T-t \right)-\alpha\beta_{2}\left( Y\left( T \right)-Y\left( t \right) \right)+\gamma\int_{t}^{T} dh(s)\ldots\left( 18 \right)$$

Where $edlev\left( T \right)$ may be extracted from Bjerksund (1991)[145] and Merton(1971) [120] as following:

$$edlev\left( T \right)=e^{-{k\beta}_{2} \left( T-t \right)}edlev\left( t \right)-\left( 1- e^{-{\alpha\beta}_{2} \left( T-t \right)} \right)EdLev+\gamma e^{-{\alpha\beta}_{2}T} \int_{t}^{T} e^{\alpha\beta_{2}S}dh\left( s \right)\ldots\left( 5 \right)$$

Substituting with equation $\left( 5 \right)$ in the left side of the equation (18), it will be easy to get equation $\left( 6 \right)$:

$$e^{-{k\beta}_{2} \left( T-t \right)}edlev\left( t \right)-\left( 1- e^{-{\alpha\beta}_{2} \left( T-t \right)} \right)EdLev+\gamma e^{-{\alpha\beta}_{2}T} \int_{t}^{T} e^{\alpha\beta_{2}S}dh\left( s \right)-edlev\left( t \right)={\alpha\beta}_{2}EdLev \left( T-t \right)- {\alpha\beta}_{2}(Y\left( T \right)-Y\left( t \right)+\gamma\int_{t}^{T} dh\left( s \right)$$

By rearranging, we can get ${\alpha\beta}_{2}(Y\left( T \right)-Y\left( t \right)$ as following:

$${\alpha\beta}_{2}(Y\left( T \right)-Y\left( t \right)={\alpha\beta}_{2}EdLev \left( T-t \right)+\gamma\int_{t}^{T} dh\left( s \right)-e^{-{k\beta}_{2} \left( T-t \right)}edlev\left( t \right)+\left( 1- e^{-{\alpha\beta}_{2} \left( T-t \right)} \right)EdLev-\gamma e^{-{\alpha\beta}_{2}T} \int_{t}^{T} e^{\alpha\beta_{2}S}dh\left( s \right)+edlev\left( t \right)$$

By dividing the sides by ${\alpha\beta}_{2}$ and rearranging we get $Y\left( T \right)$ as follows:

$$Y\left( T \right)=Y\left( t \right)+EdLev \left( T-t \right)+\frac{1}{{\alpha\beta}_{2}}\gamma\int_{t}^{T} dh\left( s \right)-\frac{1}{{\alpha\beta}_{2}}e^{-{k\beta}_{2} \left( T-t \right)}edlev\left( t \right)+\frac{1}{{\alpha\beta}_{2}}EdLev\left( 1- e^{-{\alpha\beta}_{2} \left( T-t \right)} \right)-\frac{\gamma}{{\alpha\beta}_{2}} e^{-{\alpha\beta}_{2}T} \int_{t}^{T} e^{\alpha\beta_{2}S}dh\left( s \right)+\frac{edlev\left( t \right)}{{\alpha\beta}_{2}}\ldots\left( 6 \right)$$

**Appendix (5)**

**Calculate the sensitivity of assets to the economic geography factor**

To consider the sensitivity $\beta_{11}$ of the marginal yield of a specific asset (for example, agricultural, capital asset) toward the external factors 1,2, 3,…, $i$ (sub-determinants). We will consider the distance from core economies in this case. To do that, we may regress this yield toward the distance from core economies. For example, if we want to estimate the sensitivity of marginal yield of agricultural expansion toward economic geography, we should run a panel regression between the vector of marginal yield of each agricultural expansion toward the vector of distance from core economies.

${Ar}_{jt}=IC+\beta_{11}X_{jt}+\tau Q_{j}+{FIX}_{j}+Error$

Where ${Ar}_{jt}$ is a vector of marginal yields of agricultural expansion for agricultural sectors $j^{'}s$ at time $t$.

$IC$ is the intercept.

$\beta_{11}$ represents the sensitivity coefficient of the agricultural expansion.

$X$ is a vector of the distance of each agricultural asset from core economies. After calculating $\beta_{11}$, you can easily multiply it by other Beta’s ( $\beta_{11}\times\beta_{12}\times\beta_{13}\times\beta_{1i})$ , as we showed in section 3.1.1.

$Q_{j}$ is a vector of fixed variables representing sector characteristics.

$\tau$ the regression coefficient for sector characteristics.

${FIX}_{j}$ the fixed effect of the agricultural sector.

$Error$ represents the random error term.

1. The value of $ar\left( T \right)$ may be extracted from Merton(1971) and Bjerksund (1991) [145]

$$ar\left( T \right)=e^{-k\beta_{1}\left( T-t \right)}ar\left( t \right)-\left( 1- e^{-k\beta_{1}\left( T-t \right)} \right)Ar+\sigma e^{-k\beta_{1}T} \int_{t}^{T} e^{k\beta_{1}S}dZ\left( s \right)\ldots\left( 5 \right)Type equation here.$$

 [↑](#footnote-ref-1)
